# Post-discharge “continuum of care” clinical pathway for persons with severe neurodisabilities—qualitative research to assess its concept and practicality after implementation

**DOI:** 10.3389/fneur.2025.1552692

**Published:** 2025-09-29

**Authors:** Stephanie Reichl, Romy Pletz, Aukje Bartsch-de Jong, Nuria Vallejo, Tatjana Groß, Andreas Bender, Thomas Platz

**Affiliations:** 1 Neurorehabilitation Research Group, Medical Faculty, University of Greifswald, Greifswald, Germany; 2 Schönklinik Bad Aibling, Harthausen, Germany; 3 Therapiezentrum Burgau, Burgau, Germany; 4 medbo Zentrum für Neurologische Rehabilitation am Bezirksklinikum Regensburg, Regensburg, Germany; 5 Department Neurorehabilitation, Medical Faculty, University Augsburg, Augsburg, Germany; 6 Medical Faculty, Ludwig-Maximilians-Universität München, München, Germany; 7 BDH-Klinik Greifswald, Institute for Neurorehabilitation and Evidence-based Practice, An-Institut University of Greifswald, Greifswald, Germany

**Keywords:** community-based neurorehabilitation, clinical pathway, interdisciplinary teamwork, continuum of care, trans-sectoral cooperation

## Abstract

**Introduction:**

The aim of this study was to reflect the appropriateness and practicality of an evidence- and guideline-based clinical pathway (CP) for the intersectoral support of community-based neurorehabilitation of severely affected neurological patients requiring home-based intensive care nursing, early after its regional implementation in the Federal State of Bavaria, Germany. The CP is designed to support ongoing functional progress, with specialists from three regional neurological early rehabilitation centers (NER) providing rehabilitation expertise shared with healthcare professionals in the community across Bavaria, using a person-centered, individualized approach.

**Methods:**

Qualitative exploratory study design: Semi-structured interviews with three NER-based regional outreach teams (ROFTs), followed by a multi-stage qualitative analysis and interpretation of their responses.

**Results:**

Three group interviews were conducted with a total of 10 ROFT members. A total of 304 unique responses (i) were documented. Based on their experience, the teams reported numerous healthcare-related barrier observations (*i* = 69) and consequently, negative expectation reflections regarding the care situation in the community (*i* = 10). Regarding their outreach activities, the team’s observations predominantly indicated that their interventions were successful (*i* = 12). Nevertheless, negative expectation reflections regarding the CP’s implementation prevailed for both medical aspects (*i* = 27) and networking (*i* = 41).

**Conclusion:**

Although the CP was not challenged conceptually by the field experience, the teams implementing the intersectoral collaboration faced major challenges with the continuum of care approach. Most importantly, a lack of, and variability in, qualified therapeutic resources, as well as the fact that multiprofessional team approaches were not established as a healthcare standard in the community, were noted. While the research findings support the need for a situation analysis and targeted implementation efforts, they also indicate the potential for such a hybrid collaborative center- and community-based healthcare approach for a clientele with highly specialized healthcare needs.

## Introduction

As the leading cause of overall disease burden worldwide, with increasing global disability-adjusted life-years (DALY) counts (1), effective rehabilitation strategies for disorders affecting the nervous system are needed. Although post-acute inpatient rehabilitation is frequently sufficiently established, the situation for a “continuum of care” approach in the community is frequently less well established and more variable, especially for people with more severe and complex neurological conditions and those living in rural areas (2). Despite best efforts for patients with severe neurological disorders, weaning from mechanical ventilation during inpatient early neurological rehabilitation (NER) is unsuccessful in approximately 35% of patients, and even more frequently, i.e., in approximately 46% of patients with a need for a tracheal cannula (TC) in the acute stage of disease, it is still needed at discharge, mainly due to severe dysphagia with risk of aspiration (and consecutively pneumonia) (3). When patients no longer require acute inpatient treatment and have short-term rehabilitative goals to be achieved, patients with TC and/or ventilation are discharged from the inpatient setting. To guarantee the required care for these patients in a community setting, home-based specialized intensive care nursing (HSICN) services have been developed in Germany. HSICN provides care for patients either directly in their home environment or in specialized residential groups. In Germany, the number of out-of-hospital ventilated patients has increased from 5,000 cases in 2005 to 15,000 cases in 2018 (4). In this scenario, people with more severe and complex neurological conditions and potential for further long-term recovery after discharge from NER frequently receive their healthcare from professionals with only limited, if any, qualifications and experience in neurorehabilitation. Furthermore, in the community, caregivers usually work mono-professionally and are not networked. Consequently, basic healthcare and medical support are established, while needs for continued neurorehabilitation to make use of any potential for long-term recovery are not (sufficiently) met.

This is where the innovation project OptiNIV (Optimization of post-hospital intensive care for neurological patients) (5) comes in. In this project, regional outreach follow-up teams (ROFTs), consisting of doctors, nurses, and therapists from three participating regional NER centers, accompany the patients and provide the community service providers (HSICN, therapists, physicians) with individualized person-centered care recommendations as neurorehabilitation experts over the course of 1 year post-discharge, aiming to support functional recovery and reduce the need for HSICN. Specifically, three NER centers serve as a basis for ROFTs to cover the Free State of Bavaria, a state in the southeast of Germany. With an area of 70550.19 km^2^, it is the largest German state by land area, comprising roughly a fifth of the total land area of Germany. With over 13 million inhabitants, it is the second most populous German state. The innovation project OptiNIV can thus be regarded as a “blueprint” for the set-up of similar intersectoral healthcare organizations with broad regional coverage, offering high-level expertise to the community healthcare system while implementing an individualized, person-centered approach.

In this project, this “new form of care” (NFC) is compared to standard care, with the primary outcome being “weaning from mechanical ventilation” or the possibility of “dispensing with the use of a TC” within 1 year after discharge from NER. For the NFC as a “continuum of care” approach, the ROFTs not only promote medical care regarding mechanical ventilation and TC management but also initiate a multiprofessional approach and implement an individually specific, yet comprehensive, neurorehabilitation approach based on their expertise. To support this activity, a guideline- and evidence-based clinical pathway (CP) for the co-care of severely neurologically affected patients was developed for use by the ROFTs. The CP outlines medically meaningful and necessary content for a person-centered neurorehabilitation approach with reference to multiprofessional interdisciplinary collaboration.

CPs are documented instruments that provide multidisciplinary teams with structured recommendations for treating patients with specific clinical conditions. They include various clinical target areas and the professions involved in patient care. The best available current and valid evidence forms the content basis of the CP and thus supports the evidence-based achievement of current treatment goals (6). CPs have the following characteristics: first, they provide a structured multidisciplinary care plan; second, they promote the implementation of evidence or guidelines in local structures; third, they describe the individual treatment steps for a disease; fourth, they contain time frames for criteria-based progress documentation; and finally, the treatment of a clinical condition should be standardized (6, 7).

**Figure 1:**
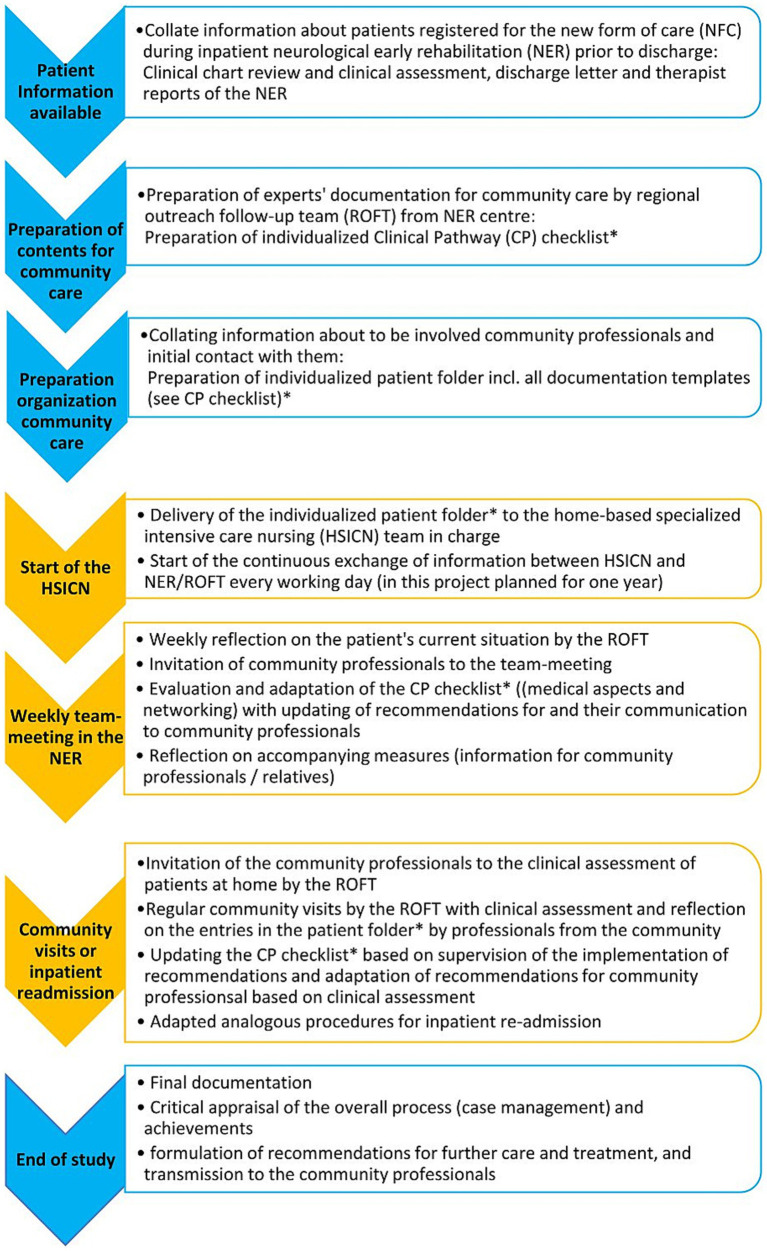
Conceptual sequence of the clinical pathway. Blue—preparatory steps in the inpatient setting (and related to end of study participation, i.e., after 1 year); yellow—implementation steps in the community setting; NFC—new form of care; NER—neurological early rehabilitation; ROFTs—regional outreach follow-up teams; CP—clinical pathway; HSICN—home-based specialized intensive care nursing. *The original patient folder documents, including the checklist, can be provided by the author if readers are interested.

The CP depicted in Figure 1 represents a conceptualization of this approach for the outpatient follow-up of severely neurologically affected patients with a need for home-based intensive care and provides tools in the form of documentation aids for its implementation. In doing so, the CP and its working aids consider the individually relevant clinical content, as well as the need for interprofessional coordination and the provision of information to all involved as necessary components of care. For these purposes (individual clinical, collaboration, information), the CP does not prescribe specific implementations but provides a matrix of possible relevant aspects that can be assessed and documented on an individual basis, together with contact details and healthcare recommendations for all involved, especially the person with the severe neuro-condition receiving treatment, healthcare professionals, relatives or legal guardians, providers of technical aids, and health insurance contact persons. In this sense, it can be understood as a problem-specific conceptualization of “case management.” It is assumed that the knowledge and the benefit of this conceptualization, together with the accompanying working aids, will significantly support the implementation of individualized, person-centered, community-based neurological rehabilitation of neurologically severely affected patients.

The contents of the CP were explicitly presented in a patient folder (for details see the “Methods” section), individually prepared and adapted for each patient. The ROFTs were tasked with implementing the CP individually and providing guidance to community-based professionals involved in the individuals’ healthcare for its practical application.

Within the framework of semi-structured interviews with ROFTs, features of the CP and its working aids were to be reviewed for appropriateness and practicality in this qualitative research project after implementation by the ROFTs.

As the research background is a regionally comprehensive healthcare project (OptiNIV) covering a large federal state of Germany with both urban and rural areas, its results might well be transferable to other healthcare situations, at least in high-income countries; the findings of this qualitative research are of interest to a variety of stakeholders including healthcare professionals of NER facilities and the community, persons with severe neurodisabilities, healthcare insurances, and politicians.

## Methods

### Study registration, ethical approval by the institutional review board, and informed consent

The study protocol received ethical approval from the ethical committee of the University Medicine Greifswald (Reg. No. BB 153/22) in November 2022.

All participants (interview partners) were provided with oral and written information about the qualitative research project and gave written informed consent before the interviews were held.

The main trial, OptiNIV, has been registered in the German Clinical Trials Register (DRKS) since January 18, 2022, with the ID DRKS00027326 (5).

### Clinical pathway documents

The CP’s implementation was supported by working aids including a patient folder as central element to be used in patients’ homes for shared interprofessional documentation. As its components help to understand the concept of the CP, its constituents will be described next.

It begins with compiled documents such as a copy of a living will and the discharge reports of the therapy professions (physical therapy, occupational therapy, speech and language therapy) from the NER. This was followed by work aids in blank format for completion, such as contact lists of the responsible persons for each profession (GP/physician specialist, HSICN, therapy professions, ROFT incl. Case manager, relatives/(professional) carers/health insurance provider/auxiliary aid provider), protocols for the ROFT-led team conferences, TC weaning protocol, ventilation weaning protocol, and positioning/mobilization protocols. They are supplemented by therapy documentation sheets for the therapeutic professions, the list of therapeutic appliances and aids to show the status of care, and a training log for training provided by the ROFT to HSICN members and therapy professionals.

This is followed by the core element of the CP checklist with eight treatment goal fields: (A) Independent breathing, (B) Independent saliva control and oral feeding; (C) Positioning and mobilization, (D) Alertness/awareness; (E) Perception/cognition/communication; (F) Sensorimotor functions and activities; (G) Emotional wellbeing; (H) Other specific health aspects.

The final section covers documentation of networking and organizational issues: (1) Interdisciplinary training, (2) Interdisciplinary teamwork, (3) Information for affected persons and relatives, (4) Facilitator and barrier factors, (5) Provision of assistive devices.

These forms were then to be used to indicate the current clinical status, specific next treatment goals to be achieved, and specific recommendations made for the various medical professions involved in the community.

### Study design

As part of the qualitative research, a focus group interview-based theory was sought to be derived for the best expression of the CP and implementation guidance. In doing so, we adopted a post-positivist epistemological lens (8). The CP is intended to support the implementation of evidence-based practice recommendations [based on quantitative research (6)] in the project. This requires contextualization (9), the success of which the qualitative research approach was considered instrumental in promoting. The research perspective was not only the project context itself, but in addition, a sustainable conceptualization of the CP was sought that could also promote transfer of the NFC into standard care in Germany (and potentially elsewhere). In this respect, the perspective of those implementing the CP in clinical practice in the project was used to modify the theoretical (and more generally valid) concept of the CP. Therefore, the research can also be considered a “grounded theory” approach (10).

### Participants

#### Eligibility criteria

In terms of purposive sampling, only multiprofessional teams that had practical experience in implementing the CP in the community were interviewed and served to promote an appropriate operationalization and contextualization of the CP, to harmonize it across teams (and hence contexts), and thus to further support the standardization of the NFC.

#### Recruitment

Three independent ROFTs of the OptiNIV project met the criteria. In each multiprofessional team (ROFT), medical doctors, nursing, and therapeutic staff* are involved. The interviews were conducted with each team as a small group (three to four people with different professional backgrounds) (focus group interviews). Thereby, the integration of differential experiences and perspectives, and hence equity and diversity of the information collated, was sought to be promoted.

### Study procedures

ROFTs were visited for a 1–1.5-h on-site interview (at the ROFT’s facility) after they had approximately 2 months of field experience with the CP. The interviews were followed by a multi-stage qualitative analysis and interpretation process including transcription of the interviews, initial review of the transcripts, deduction of emerging response categories and creation of corresponding “codes” (with anchor examples), identification of unique interview responses in the transcripts of the interviews (and their code category), summarizing the contents of unique responses for these categories, derivation of emerging themes (including a “conceptual map”), and a conceptual synthesis of the findings in terms of theory on how best to express the CP and its implementation guidance. The results were presented to project stakeholders for consultation. Thereafter, a final version of the CP and associated documentation aids was made available for further implementation.

### Data collection, conducting the focus group interviews

#### In advance

Basic explanations of the goal, content, and procedure, as well as the desired active participation of all interviewees, were provided, including the opportunity to clarify questions. Additionally, the interviewees were verified, written informed consent were obtained, and the interviews were scheduled.

#### Interviews

The interviews were conducted on site at the regional outreach centers (ROCs) by two researchers (SR and TP), following an interview protocol and audio recording. Interviews took place from November 9 to November 11, 2022.

At the beginning, the goal, content, and procedure, as well as the desired active participation of all participants, were once more explained, and the opportunity to clarify questions was provided. The characteristics of the interview participants (age decade, sex, occupation, years of work experience in neurorehabilitation) were then documented. Thereafter, the interview questions were systematically asked (qualitative research) (Table 1). At times, more detailed information was obtained using prespecified prompts (Table 1). The interview concluded after all questions were addressed.

**Table 1:** Semi-structured interview script.

Dimension	Question
Experience	**First experiences** with the CP in practice as an open question (“ice breaker”) Elicitation of **aspects of care that are (basically) not feasible** in the care situation (care system)
CP contents	CP—Specified **treatment goals** (adequate and how specifically applicable? What is missing, if any?) CP—Specified **dysfunction(s) in each target domain** (adequate and how specifically applicable? What is missing, if any?). CP—Formulation of the question **of consideration of functional disorders in everyday life** (adequate and how specifically applicable?) CP—Possibility of documenting specific **recommendations** (adequate and how specifically applicable?) CP—Formulations for the topic area of **interdisciplinary training** (adequate and how specifically applicable?) CP—Formulations for the topic area of **interdisciplinary teamwork** (adequate and how specifically applicable?) CP—Formulations for the topic area **information for affected persons and relatives** (adequate and how specifically applicable?) CP—**patient’s notebook** (form and content adequate, and how prepared useful for interprofessional collaboration?) CP—Are there **other aspects** not already discussed that would be important to consider for updating the CP?
Prompts	Please tell us more about it Can you describe this in more detail? What would be an example of this? Can you explain your answer a bit more (or in more detail)? What does (e.g.) “not good” mean?

### Data analysis and outcomes

As a first step, the interviews were transcribed verbatim. All interviews were digitally recorded and transcribed offline by RP, and the transcripts were independently validated by SR. Transcriptions were formatted in three columns: column one contained the ROFT code and the question addressed, column two contained line numbers and text, and column three contained the coding of interview partner responses.

Next, an initial review of the transcripts was conducted. The initial review served as an overview of the interview results and as preparation and orientation for the subsequent qualitative analysis steps. Diverse aspects of the contents of responses (“codes”) were deduced (emerging *post hoc*) from the interview material, and a codebook was created (TP and SR). These codes were intended to represent different distinguishable thematic aspects of interview content that may be relevant for the conceptualization of the clinical pathway and its implementation, as well as the design of the practice aids, and may recur with different expressions and wording across individuals or interviews. Codes were described in terms of content, assigned a 1–3 word name and a code number, illustrated in more detail with plain-text anchor example(s), and compiled in a codebook.

In the next step, the specific responses from interview partners were identified. Two independent assessors (RP and SR) evaluated the transcribed interview text for unique responses, which were marked together with the appropriate code (i.e., the code name and number, as well as the text line [beginning of the related transcribed text]) documented in one column, with the corresponding text section marked in the an adjacent main column of the transcript. The line number of the text can be taken as an independent variable, and the unique response with its related code as a dependent variable. When the ratings of the two independent assessors did not agree, the coding was discussed in group meetings with a third rater (TP) to reach a final consensus. For each specified content aspect (i.e., code), the content of the unique responses given by interview partners was then summarized. Summarized content (per code) was collated within “themes,” and their logical relationship was graphically illustrated using a “conceptual map.” Subsequently, a conceptual synthesis of the results and their relevance for the theory for the best expression of the CP, with framework recommendations and implementation aids, was deduced. Based on the contents across themes, implications for the conceptualization of the clinical pathway and its implementation, as well as for the design of the documentation aids, were derived, and adaptations performed. The results were presented to OptiNIV project stakeholders for consultation, after which a final version of the CP and documentation aids was made available for further implementation.

### Data management

The digital audio files of the interviews were stored on two project-specific Veracrypt-encrypted hard drives (original & copy) in restricted-access workrooms of the neurorehabilitation research group (NRG) of the University Medicine Greifswald for the duration of the OptiNIV project (until 07.2025) and will be deleted afterward. The transcribed text contains only anonymous information and will be stored longer for later secondary analyses by the NRG. Furthermore, the ratings and markings of the raters were entered into the text files (1 file per rater and a file for agreed (final) codings).

### Statistical analyses

As the research was planned as qualitative, no hypothesis-testing statistical analysis was intended. Although exploring thematic content aspects emerging from interview responses was the focus of the study, descriptive statistics on the type and frequency of codes, clustered into emerging themes, were provided as additional aid for interpretation. *A priori* sample size collection was not indicated (no hypothesis testing).

## Results

### Development of coding and categorization

The three interviews were conducted with 10 individuals (three physicians, two speech and language therapists, two nurses, two study assistants, one occupational therapist) from the three ROFTs, reflecting diversity of age, profession, and past experience in neurorehabilitation. Details about the participant description are given in Table 2.

**Table 2:** Description of study participants (*n* = 10).

Participant	Sex	Age decade	Profession	Professional years in neurorehabilitation
1	f	2	Physician	3
2	f	1	Speech and language therapist	7
3	f	2	Study assistant	0
4	f	4	Physician	9
5	m	3	Study assistant	2
6	m	3	Occupational therapist	12
7	f	4	Nurse	10
8	f	3	Nurse	16
9	f	1	Speech and language therapist	1
10	f	2	Physician	4

Sex: f = female, m = male; age decade: 1 = 18–29 years, 2 = 30–39 years, 3 = 40–49 years, 4 = 50–65 years.

During the first step of qualitative data analysis of the interviews, 34 distinguishable emerging content aspects (codes) were identified and operationalized in a code manual and grouped into five thematic fields with 13 thematic code categories, as shown in Table 3.

**Table 3:** Overview of the 52 categories, grouped into five thematic fields, 13 code categories, and 34 subcategories.

Thematic field	Observation—community	Σ 77
*Thematic code category*	*Positive non-systematic casuistic observations*	**4**
Code subcategories	Current situation	2
	Development over time	2
*Thematic code category*	*Negative non-systematic casuistic observations*	**1**
Code subcategories	Current situation	0
	Development over time	1
*Thematic code category*	*Facilitating aspects*	**3**
Code subcategories	Healthcare	1
	Networking	2
*Thematic code category*	*Barriers*	**69**
Code subcategories	Nurses	15
	Therapists	14
	Physicians	6
	Therapy, technical aids, medical products	17
	Medication	5
	Networking	12
**Thematic field**	**Observation—outreach intervention**	**Σ 87**
*Thematic code category*	*Intervention success*	**12**
Code subcategories	Healthcare	2
	Networking	10
*Thematic code category*	*Intervention failure*	**6**
Code subcategories	Healthcare	1
	Networking	5
*Thematic code category*	*Aspects of patient folder*	**54**
Code subcategories	Positive	12
	Negative	42
*Thematic code category*	*Interpersonal/emotional reaction*	**15**
Code subcategories	Positive	11
	Negative	4
**Thematic field**	**Expectation—community**	**Σ 13**
*Thematic code category*	*Expectation reflection, healthcare*	**13**
Code subcategories	Positive	3
	Negative	10
**Thematic field**	**Expectation—outreach intervention**	**Σ 105**
*Thematic code category*	*Expectation reflection, clinical pathway/medical aspects*	**28**
Code subcategories	Positive	1
	Negative	27
*Thematic code category*	*Expectation reflection, clinical pathway/networking*	**60**
Code subcategories	Positive	19
	Negative	41
*Thematic code category*	*Understanding of its role by ROFT*	**17**
Code subcategory	Regional outpatient follow-up team (ROFT)	17
**Thematic field**	**Familiarity aspects—related to ROFT team member**	**Σ 22**
*Thematic code category*	*Regional outpatient follow-up team (ROFT)*	22
Code subcategories	Reflection of treatment goal	2
	Differential capturing of functional deficits	1
	Consideration of functional deficits in everyday life	2
	Differential generation of recommendations	4
	Interdisciplinary education and training	5
	Interdisciplinary teamwork	6
	Context-based risk management	2

Explanations: numbers indicate the number of entries, i.e., unique responses from interview partners within the respective code (sub-)categories.

The five thematic fields included either aspects of healthcare in the “Community” (i.e., independent of the NFC) or those made specifically related to the “Outreach Intervention” (NFC). In addition, for both of these domains, interview partner responses were partially based on observations made or on expectations related to either healthcare in “Community” or for the implementation of the “Outreach Intervention.” Furthermore (fifth thematic field), interview responses could indicate the degree of familiarity of ROFT members with aspects specifically related to the healthcare addressed and the implementation of the CP.

The 12 thematic code categories include, for example, observed “facilitators” and “barriers” in the community care situation, but also reflections on expectations regarding various aspects of CP, such as the medical aspects, networking, or the ROFTs’ understanding of their role.

### Thematic processing of the transcript statements

The interview transcripts were then analyzed in detail by two independent raters (SR and RP), and a total of 304 specific, unique responses from the interview partners were identified. An overview of sample statements in the English-translated version of the responses for all thematic code categories is shown in Tables 4a–d. For the original German transcripts, examples of these specific, unique responses are provided in a Supplementary Table S1.

**Table 4a:** Overview of sample statements from all code categories [English translation].

**Thematic field**	**Observation—community**
*Thematic code category*	*Positive non-systematic casuistic observations*
Code subcategory “Current situation”—sample statement	“One time we briefly saw from an occupational therapy perspective that the daughter and grandchildren were making materials for fine motor work.” (T)
*Thematic code category*	*Negative non-systematic casuistic observations*
Code subcategory “Development over time”—sample statement	“And I have to say that I was also extremely surprised that we had the two patients who died so quickly. I did not expect the first 2 weeks to be so critical. I thought the critical phase would come later. But I did not expect it to be so early, so quickly.” (P)
*Thematic code category*	*Facilitating aspects*
Code subcategory “Networking”—sample statement	“You can also tell within the team that the colleagues, they sat together over coffee and exchanged ideas.” (N)
*Thematic code category*	*Barriers*
Code subcategory “Nurses”—sample statements	“What can they? What do they want? Then you get the answer: “Yes, I’ve been in this job for 20 years now.” But you realize that nothing has changed there in 20 years. These are very different aspects.” (N) “But there is one topic, transcranial direct current stimulation […], which is certainly not even feasible in some neurorehabilitation, where you certainly cannot think about it in the community. Because there is neither a consultant who could supervise it, nor a therapist or nurse who could apply it.” (T) “So I often see in the field of HSICN that the main mandate to act is to ensure that the patient is SAFE, CLEAN, SATIATED […] But it’s not the case that these ADVANCED rehabilitative areas, if it’s not someone who really enjoys working with the person they are caring for, then it’s a case of service to rule.” (T) “Because we naturally have a shift service with different people who also change within it, it’s very difficult to map consistency.” (T)
Code subcategory “Therapists”—sample statements	“I think I would add one more thing about therapists or therapies. Yes, one of the challenges is to find at all therapists who in particular can provide care in rural areas, let alone suitable therapists who have already worked with neurorehabilitation. Perhaps also, especially in the case of speech and language therapists, we see that there is simply one practice, for example, where one speech and language therapist can unblock and as soon as she is absent, other things happen in therapy. Which does not necessarily have to be wrong, but certain processes or certain goals simply cannot be pursued for weeks because there simply is not the right staff on site or, for example, the HSICN leaves it entirely to the speech and language therapists or vice versa.” (T)

**Table 4a:** (Continued)

*Thematic code category*	*Barriers (Continued)*
Code subcategory “Therapists”—sample statements (Continued)	“I have also noticed that tracheostomy tube management is handled differently from hospital to hospital. There has to be a delegation from the doctor to the speech and language therapist and if the hospital does not do it, then the speech and language therapist is not allowed to do it at all. So just because you are a speech and language therapist does not mean that you are automatically authorized to carry out tracheostomy tube management.” (P)
Code subcategory “Physicians”—sample statements	“And above all, because they have no opportunity to seek help from community doctors. Because the doctors in the community often cannot cope at all with the patients, with the clinical picture of this patient, that we discharge in such a serious condition.” (P) “The GP as such is still a generation that finds it difficult to deal with such patients.” (N)
Code subcategory “Technical aids”—sample statements	“We notice that patients often receive the aids very late. They are often refused, and the status is often not clear at all in the shared flat or is difficult to ask about.” (T) “One big thing that we are regularly involved in and are now trying to get a foot in the door is the provision of aids. We notice that patients often get their aids very late. They are often rejected, and the status is often not clear at all in the shared flat or is difficult to ask: “What’s the situation with the hoist? Has it been approved? Has it even been approved, or has it been rejected again? Who’s taking care of it right now?”“(T) “Despite the fact that we actually have a long lead time of three to 4 weeks here at the hospital. And the fact that we have already initiated everything that’s necessary. That, nevertheless, a lot of things are not on site in the end.” (P)
Code subcategory “Medication”—sample statement	“Or drugs that are regularly used in the hospital setting are canceled in the community setting because they have to be paid for by the patient.” (N)
Code subcategory “Networking”—sample statement	“Exchange with the therapist, unless he happens to be there the hour before, does not work. Of course, the exchange, they do their own documentation in the patient chart in the HSICN.” (P) “With patient conferences, the way we can organize it here in the hospital, that’s not possible. Besides, our therapists here in the hospital, if we schedule the patient for a conference now, they get paid. The therapist in the community either takes the patient hour, which would be quite dramatic, or he takes it from his free time, but why?” (P)

HSICN, home-based specialized intensive care nursing; GP, general practitioners; T, therapist (occupational therapist, speech and language therapist); N, nurse and study nurse/assistant; P, physician.

**Table 4b:** Overview of sample statements from all code categories [English translation].

**Thematic field**	**Observation—outreach intervention**
*Thematic code category*	*Intervention success*
Code subcategory “Healthcare”—sample statements	“We really have one patient with us who is making progress after progress, who was simply up against the wall in the hospital […] he stagnated, did not make any further progress. And now he’s making progress in the HSICN.” (N) “In one patient, ACV was introduced, i.e., this Above Cuff Vocalization, via subglottic suctioning. Yes, that was very nice to see, because the team was interested in it and was not yet aware of this possibility of actually clinical treatment methods. So they were familiar with it, the speech and language therapist, the occupational therapist, the HSICN team were there and the respiratory therapist from the shared flat. They took it very well and were amazed that the patient, who had never spoken before, was able to speak.” (T)
Code subcategory “Networking”—sample statement	“And I have to be honest, I also had the impression that the conversation with the relative led to us having more relationships with the shared flat again anyway. They also felt that it was a relief that we had invested in them. And that’s where the cooperation or something has grown again.” (P)
*Thematic code category*	*Intervention failure*
Code subcategory “Healthcare”—sample statement	“Or special things to reduce secretions. If it’s in drops, it’s prescribed, in my case it was gastrozepine, you can have it in drops, but if it’s IV, it’s not prescribed. So there are, they are pushed up against the wall so much, the residential groups. So even with our recommendations as well, I found that negatively impressive.” (N)
Code subcategory “Networking”—sample statement	“Yes, I did not know the patient and that’s exactly how the situation developed. It wasn’t possible for us to get into the team. There was simply no interest, exactly, from any profession. Neither the therapists, nor the nursing staff, nor the doctor were interested in us being allowed to act as a team member. There was more of a feeling that, as “P” said, it was more about remaining a close-knit circle, for whatever reason.” (T)
*Thematic code category*	*Aspects of patient folder*
Code subcategory “positive”—sample statements	“So as an example, the clinical pathway itself is logical.” (T) “So basically, it has a concept, the system.” (P) “Nevertheless, there are important points and good points that you can work through if you have the sheet. It’s also quicker to formulate, with a cross, than to formulate the whole thing.” (T)
Code subcategory “negative”—sample statements	“So I think you often think about the question of the extent to which manual paper documentation, i.e., whether the long-term goal would of course also be whether such an exchange could not also work digitally. So that all partners can document and send the information THEORETICALLY from their therapy desk, where they work in practice anyway.” (T)

**Table 4b:** (Continued)

*Thematic code category*	*Aspects of patient folder (Continued)*
Code subcategory “negative”—sample statements (Continued)	“We have actually had the best experience in personal exchanges either on site or over the phone. You do not know […] when it will be read, by whom, what they think of it. Who takes it further.” (A) “And I think, with regard to therapy documentation again /. We see that there is often a way of documenting therapies in the HSICNs themselves. Depending on the location, depending on the therapist constellation, it was then used or not. I’ve already spoken to several speech and language therapists and they often say: “I have to document things for myself in the practice anyway. And then I have to document in the HSICN itself and so to speak again at home “. And the fact that this then matches, that the same information is in there and the whole thing works, that just seems to be a lot of effort for many. Because it’s simply not as easy to transfer one-to-one as sending around the documentation from today by e-mail, that’s not easy. And, well, that’s one of the things that the therapists also say, that we also had a part of the clinical pathway with the therapy documentation. That would be the third document into which you could write something.” (T) “Perhaps we can also say that we also wanted to deal with the issue of data protection in this regard.” (T) “And also because resources are scarce, I think it’s a very helpful tool for us and I would actually prefer to maintain and have it here. And then only give them a summary of what is really relevant for them or what is perhaps missing locally. […] To read into it and what the recommendation that comes out of it really is, that is often just a sentence or so that we would really recommend to them. And then I would only communicate the sentence and perhaps also in a telephone conversation, where you can talk about it straight away. I think it’s then simply more fertile ground to fall on.” (P)
*Thematic code category*	*Interpersonal/emotional reaction*
Code subcategory “positive”—sample statements	“And how safe relatives feel when they know they will continue to be accompanied by these people.” (N) “We have not had that experience yet. What we do experience at the beginning […] is that we are always greeted by patients who come from their own stable, they say hello, they are happy that we have come by, or they say hello back.” (N) “Yes, on the one hand it was very nice to see, because the team was interested.” (T)
Code subcategory “negative”—sample statements	“And then always in the back of your mind, this countering /. An incredible amount of money is paid for this place every month. And then these are such […] small amounts […] that it fails because of these pennies.” (N)“For example, we currently have a patient at home where the relatives are incredibly anxious and every step taken by the HSICN team, regardless of whether it is good or bad, is viewed with suspicious eyes. Ideally, every step we take should be stamped as ‘good.’” (T)

HSICN, home-based specialized intensive care nursing; T, therapist (occupational therapist, speech and language therapist); N, nurse and study nurse/assistant; P, physician.

**Table 4c:** Overview of sample statements from all code categories [English translation].

**Thematic field**	**Expectation—community**
*Thematic code category*	*Expectation reflection, healthcare*
Code subcategory “positive”—sample statements	“Giving the HSICN confidence is a big problem. I’ve always had the feeling, coming from forty years of intensive care, that when you move patients into homes, shared flats and so on, there’s a kind of giving up. And that’s a change in thinking and that’s also happened to me now. It was certainly more like that in the past, is /. But I’ve also had a rethink, so the patients we have observed now are in very good hands.” (N) “And on the other hand, there are also shared HSICN that are already at a very high level, where everything is already running smoothly, where there is a network between them, where they really work together with therapists and doctors. Where you realize that we are basically just there, not superfluous, but there’s not much more for us to do because things are going really well.” (N)
Code subcategory “negative”—sample statements	“(.) but where we are now, in XYZ, there is actually, yes, only maintenance, no deterioration.” (N) “(.) and go on to other teams with other patients who have already been discharged with pressure sores and who are lying on their backs all the time and have no possibility of positioning and no aids for positioning, there are none, so it’s very different.” (P) “So special things like that, that’s difficult, nobody out there knows that yet. So the patients who are now minimally conscious do not really go beyond the standard therapy with movement and normal care, to be honest, so subjectively they fall behind. Even if we have not had that many yet.” (T) “But the staff shortage is even more extreme in rural areas.” (P) “It’s easy to see what a speech and language therapist is, what a speech and language therapist is in that sense, or simply this swallowing therapist. Or this qualification of respiratory therapist, which would be so important in every HSICN. To see that there are things, I do not want to say “that hurt.” But you think: “If all this were there now, you could really do more for the patient, you could achieve even more.” That’s the thing, yes. That’s this negative, not negative, simply the experience that we have had, that we would not have thought that we would encounter this problem.” (N) “The implementation of high-frequency therapeutic therapy, especially speech therapy or respiratory therapy /. Because it would actually be desirable for the patient to receive care and treatment daily, or at least realistically three to four times a week. But sometimes, as I said, it’s not even possible once a week.” (T)“I think what you can also add here is that many HSICNs say of their own accord that there is also a fear of complications. Even if they say that they often feel that patients are better cared for by themselves than if they transfer someone to an acute hospital. They do not know anything about the case and are a new practitioner in this whole field. They have no contact at all with the external system in which the patient is located. And since sometimes things are changed and the patients come back and everything is different and everything is new. And sometimes they have, or it seems to them, that they have a more unstable problem or system than before. And then maybe they do not vomit any more, but then there are other problems because maybe some medication has been discontinued or things like that. So we hear that again and again.” (T)

HSICN, home-based specialized intensive care nursing; T, therapist (occupational therapist, speech and language therapist); N, nurse and study nurse/assistant; P, physician.

**Table 4d:** Overview of sample statements from all code categories [English translation].

**Thematic field**	**Expectation—outreach intervention**
*Thematic code category*	*Expectation reflection, clinical pathway/medical aspects*
Code subcategory “positive”—sample statement	“I think that’s where it’s still the most structured, because clinical reasoning is the most established in the medical service, from my point of view as a therapist, because you simply see very clearly: “Okay, I’m giving a medication, for example, and I’m hoping for this effect. And if it does not work, then I’ll stop taking it or think about taking another one.” (T)
Code subcategory “negative”—sample statements	“Well, I think it depends. It varies a lot from HSICN to HSICN. I think if you look at it, I think many have a system that works for THEM. I’m going to say that the minimum they need for day-to-day work works. For this approach, that we REALLY want to fully exploit this potential, it is of course a different question whether the system is conducive to this.” (T) “Certainly in terms of standards: when is unblocking allowed? With which cannulas, et cetera. These are often things that are not really written down. There’s a nursing manager somewhere or someone who has a concept in their head.” (P) “And the other thing is, of course, there is already one or the other. And the problem is, of course, that nobody likes duplicate documentation. But an additional problem is of course that resources are already very limited. Certainly in terms of therapists and doctors. And that they already document very little in the current situation. And that, for example, every shared flat actually has some kind of documentation sheet for the therapists. If these are not used, it will not do any good if we add a second edition.” (P) “And that’s also difficult because there are very different, I’ll call it, quality levels within a team. It’s not always easy to find out where the team is now.” (N)“The patient was hospitalized due to a cannula malposition. Had several drops in saturation that could have become really dangerous. So soon on the cusp of becoming dangerous, an intervention was necessary. And when these patients are repeatedly asked why they need a pulse oximeter, even though we are in the decannulation process in theory. In my opinion, this also belongs in the clinical pathway, that this is also a problem. Because, in the end, it hinders all parts of the clinical pathway, because we ultimately receive the block from the payer. Which we first have to process formally, even if, technically, the device is already there. But it takes up an incredible amount of space, even at the HSICN: “Yes, we have received another letter, what do we do now? Can you help us with this? That’s not easy. Maybe that can be mapped somehow. Because, as I said, it also describes processes.” (T) “Well, the therapists in the HSICN, of course they also make their documents. My experience with the nursing staff is that they are very careful. And I think the only way we could implement therapy suggestions better would be if we had more therapy hours and the nursing staff were better equipped or more staff were available. And this staff would also be better trained. They are all very good and they do everything they can. But if I was a nurse in the HSICN and I did not have a doctor at my side, I did not have a therapist at my side, I would be VERY careful.” (P) “Yes, as I said, the nursing staff are very reluctant and I understand that. They cannot take responsibility, we only take responsibility for our recommendation, but of course the relevant nurse or GP is then responsible for implementing it. And they always wait, and should wait, for feedback from the GP in charge.” (P) “So I think that a good exchange can only work through regular contact and that the team has confidence in us in the first place. That they also see that we are really available to them in an emergency. But the reality is that if there’s an emergency at the HSICN and we are not available, we cannot provide all-round emergency care. That’s why I also understand that the teams are reluctant to go too far with therapies.” (P) “I think one thing that causes a lot of anxiety is the liability issue. Who is responsible if things go wrong? If the patient, in a good case, only gets pneumonia, in the worst case dies? I could imagine that if the speech and language therapist had ordered this on an outpatient basis, everyone would immediately say: “Yes, it’s the speech and language therapist’s fault.” And of course I would not want to be in their shoes. It would be good if it could be shifted back to medical liability. It’s probably a problem constellation like that of midwives who work independently outside.” (P)
*Thematic code category*	*Expectation reflection, clinical pathway/networking*
Code subcategory “positive”—sample statements	“And the question, I think, is generally how this transfer of knowledge between therapists and the HSICN actually works. And I think a lot of it happens orally.” (T) “So I found that to be quite fruitful and I also think that an exchange at various points makes sense, perhaps not every week. But I think it makes sense to try and get everyone communicating with each other a little bit.” (T) “So I think that a good exchange can only work through regular contact and that the team has confidence in us in the first place. That they also see that we are really available to them in an emergency.” (P) “And what else we can do, and we have already done this, is to give our e-mail address to the speech and language therapists and say: “Okay, just write to us if you have any questions. Call us on this number if you have anything. If you do not know how to proceed or if there is a regression. Simply report any observations.” The HSICN does this anyway in the daily exchange, so that they can report back to us. And as I said, we also get the speech and language documentation. If we noticed something, we would report it back. And we try to support the speech and language therapists who are on site as much as possible and simply offer them a certain level of security. That they also have more confidence to take part in tracheostomy tube management and help drive it forward.” (T) “We do not know from the start whether they will cover the patients or not. We then call and ask: “Hey, do any of you have time? Do you have capacity? We need /.” And then the practice says: “Yes, we can or no, we cannot.” And sometimes you have a few unstable candidates or something. And then you can also say: “Yes, we have staff. We can support them, we can support them if advice is wanted.” (T) “Because we asked the relatives and the HSICN beforehand: “Hey, we are doing a study, do you want to take part”? And the relatives are usually very happy and very grateful. And I have not yet had a case where the relatives have said: “No, we cannot give out the document now.”” (P)

**Table 4d:** (Continued)

Code subcategory “negative”—sample statements	“And it’s often not so clear what, for example, a therapist is allowed to decide for themselves? Where does he need the GP’s consent? And these are things where weeks can go by and then nothing happens. And you just end up back where you started.” (P) “Because if you do not communicate this, the nursing service management in the residential community say: “If my girls or my people, sometimes they call it that, if they have to do more, then we will not do it.” And the threshold is incredibly low. You do not even think it, but the threshold, this REJECTION THRESHOLD, is incredibly low.” (T)“One other point that comes to mind and is important is that they have a different goal and a different way of thinking. In rehab, we are just: “How can you move forward and achieve the maximum”? And they have a much greater need for security. And that’s understandable because, I mean, we might have a bronchoscope zig zag there or whatever, an intensive care team. They just do not have that there. And that also affects the implementation of our recommendations. It’s understandable that we encounter resistance in some cases.” (P)“So if the GP, well, we also have this with another patient, if the GP then has to call the ROFT because, quite naturally, he cannot be blamed for not knowing how to set up a cough assistant because the HSICN team has requested one. Yes, but is completely overwhelmed by the situation, and the relatives naturally say: “Well, but why do not you know that now? You prescribed it.” “Yes, because that’s what the carers wanted.” And yes, that really creates a vacuum situation that is difficult to resolve.” (T) “At the same time, I have the impression that there is a need for more multidisciplinary exchange. But that the structures and the time and, of course, the funding are not there, exactly.” (P)“And I also believe that it is difficult for the relatives /. I imagine it’s a bit difficult, because as a relative I put my, again, the relative in this HSICN so that I do not have to take care by myself, because I cannot take care of it, because perhaps I’m not emotionally able to, because perhaps I’m not familiar with the issues at all. If you, maybe these relatives are then happy that the person is well protected in this HSICN. And if you then take them back on board to take care of all this clinical stuff, I think a lot of people simply block it. Because I think they tend to focus on this emotional /: “it’s still my partner, it’s not a patient,” I think.” (T)“With patient conferencing, the way we can organize it here in the clinic, that’s not possible. Besides, our therapists here in the clinic, if we schedule the patient for a conference now, they get paid. The outpatient therapist either takes the patient hour, which would be quite dramatic, or he takes it from his free time, but why?” (P)
*Thematic code category*	*Understanding of ROFT role*
Code subcategory “Regional outpatient follow-up team (ROFT)”—sample statements	“And to a certain extent, it will certainly also be the case from our side that through the communication of the clinical pathway, through the routine, we will certainly then also adapt more to the situation of the outpatient clinic. And then perhaps we will be able to communicate one thing or another differently, better, ideally.” (T)“That’s why topics like unblocking or weaning, which is also part of the project, are simply viewed with caution from the HSICN. There are many hurdles, I think. The question is a little bit, how do we work with it and how can we get into it?” (T) “Yes, of course, we are going to sort it out via the legal guardian and so on, that’s clear, that’s our job. But these are the stumbling blocks. For us clinicians, these are actually quite logical things that we simply say and expect to be done, perhaps after brief discussions. But it does not work, because there’s always something that puts the brakes on. And also in this simple structure: the speech and language therapist was informed that he should do it, but he cannot because the legal guardian has to agree to it first. Because in the hospital it was clear, because the attending physician is ultimately the one who gives or does not give the go-ahead. Because it is medically indicated or not. But outside the hospital, it suddenly looks completely different. Or the doctor has to agree to it, the legal guardian can (not?), depending on the situation.” (T) “Exactly, and I think otherwise we also have appointments with them, i.e., telephone appointments, and there’s another one coming up this week with a relative. But I think you also notice that this is also relationship work. Because I think this STUDY setting is still completely new for some people. Then the question is: “What role do we actually play in this whole thing?” I had the same feeling with the one relative I spoke to on the phone about Ms. X. Then I got the feeling that he first wanted to know what we were actually doing. Of course, the clarification happened beforehand, but this: “How does it look like in everyday life now?” It’s quite normal.” (T) “I found it very useful to have a relative in the study visit so that we could have some personal contact. We had a long discussion. We spent an hour, I’m going to say an hour, providing very basic information on the subject of dysphagia. And I had the feeling that it was very important at that moment. Because the patient has been dependent on a tracheostomy tube for three quarters of a year, or six months. And I think that until this is noted it’s important that someone feels responsible for doing this educational work. Because, I think in this, we also have to be aware that in the acute situation of neurorehabilitation, some information is simply too much and cannot be processed and absorbed at all. If you say: “Well, he’s going to get rid of the cannula, then maybe I will not really deal with it.” And later, when it’s all set and it’s such a permanent situation for the first moment, then I deal with it first and then there are lots of questions. And I do not think everyone in the community setting feels responsible, that’s my impression, to do such an intensive BASIC education. Because, I think, many people assume that the rehab has already done this or perhaps a doctor has already done it.” (T)

**Table 4d:** (Continued)

*Thematic code category*	*Understanding of ROFT role (Continued)*
Code subcategory “Regional outpatient follow-up team (ROFT)”—sample statements (Continued)	“Of course, that’s also what we do in our daily conversations. We do not just do that during the visits. But in our daily discussions, we consider how far a subglottic cannula with subglottic suction can still be used, even though a window cannula is now also possible. Whether the cannula, which has a larger outer diameter than the cannula /. That is, of course, that is now, I also feel, that is our daily work or our task.” (N) “Because an existing system is of course always difficult from the outside with someone you have seen twice to throw all your beliefs against the wall, let us say. We’ve noticed that we often get into conversations.” (T) “And that’s perhaps another point. That I’m still looking a bit for a way to not support good systems too benevolently, but also not to weaken certain structures. By making it less clear who is responsible for what, for example. If four people are already working intensively on it anyway, then the danger is always greater that if someone else comes in, there will only be more confusion and delays.” (P) “May I interject briefly. From a speech and language therapist’s point of view, I find it a bit STUPID in the sense that if I have an external speech and language therapist who goes to an HSICN, then I actually know as a speech and language therapist: “Okay, I’ll do the training for TC management et cetera PP beforehand.” And if someone were to say to me: “Excuse me, you can do further training,” I would think to myself as an external speech and language therapist: “Yes, I know that. I know the training courses that are on offer and I already know that there are training courses.” So I might find that a bit difficult. It would be different if I were to suggest: “Hey, we are coming as a clinical team and we’ll give you the training.” I think I’d be more likely to accept that than if you told them about the offer, which everyone would do on their own initiative anyway. Because, for us speech and language therapists, it’s a standard procedure that we have to do two or three training courses a year, simply to keep up to date.” (T) “We mentioned that earlier, I do not know HOW. In other words, how we should put it into practice. And I also think it’s THRILLING that in this CAREFUL clinical setting we have to recommend someone who is really working alone on patients outside. I would also feel like I was stepping on toes if someone from outside said: “Oh, in the hospital /. You can do it this way and that way, why do not you do it?” (P) “But actually, if there’s an emergency in the HSICN, we are not available, we cannot provide all-round emergency care.” (P)

ROFT, regional outpatient follow-up team; T, therapist (occupational therapist, speech and language therapist).

HSICN, home-based specialized intensive care nursing; ROFT, regional outpatient follow-up team; T, therapist (occupational therapist, speech and language therapist); N: nurse and study nurse/assistant; P: physician.

**Table 4e:** Overview of sample statements from all code categories [English translation].

**Thematic field**	**Familiarity aspects—related to ROFT team member**
*Thematic code category*	*Regional outpatient follow-up team (ROFT)*
Code subcategory “Reflection of treatment goal” sample statements	“I can agree with that right away. The aim with one patient is really to extend the speaking valve times and plugging times. For the other patient, we only need to suction eleven times a day instead of twelve. Definitely. So it’s like this, you can break it down into operational goals and strategic goals as you like. But maintaining the status quo is also a goal.” (N)
Code subcategory “Differential generation of recommendations” sample statements	“The challenge is to find out, and I think you described this quite well in advance, which documents make sense and HOW. And what can you really contribute for this one case, exactly.” (T) “Because we just gave this recommendation: “Do this, because unblocking is still difficult.” But to establish swallowing: “Please do this.” “Yes, how does that work?” “Okay, we’ll offer you an appointment for us to come by. Very simple technical story but we’ll do it together once.” And we were told that they would do it now.” (T)
Code subcategory “Interdisciplinary education and training” sample statements	“I think we have already thought about which training courses make sense. We also have two acute issues. So I think now with the speech and language therapists, I could even classify some phone calls as training. Because we often discuss very specific cases or general topics. For example, when we talk about why my patient should prefer to eat with an unblocked tracheostomy tube. It has to be said that there’s a lot going on, even in the weekday calls. On the other hand, we have already planned to carry out a training course in a shared flat for the implementation of unblocking on ventilation. And I think it’s good that this is documented somewhere, who is involved and who knows about it. On the other hand, I do not think the shred flat would look it up, but rather just ask the team: “Who was involved in the training?”“(T)
Code subcategory “Interdisciplinary team work” sample statements	“And it does not make sense to write in all nursing staff, for example, and we also see data protection problems.” (N)
Code subcategory “Context-based risk management” sample statements	“And if we give a treatment plan for a situation that is not at all predictable, we can say: “Expand plugging times.” Then they may find themselves in an emergency situation because we have given them the impetus to do so. And there’s also a lot of uncertainty, I understand, because of course they are well protected here, I have my resuscitation team here, if something goes wrong, no problem. And out there, they have to wait until the emergency doctor arrives.” (P)

ROFT, regional outpatient follow-up team; T, therapist (occupational therapist, speech and language therapist); N, nurse and study nurse/assistant; P, physician.

These responses were marked in the transcripts along with the interview partner’s profession and the corresponding manual-based codes. In line with the principles of equity and diversity in data collection, all responses were considered informative.

Emerging from the 304 unique interview responses and summarizing these for the specified content aspects (“codes”), 25 summary statements could be deduced from the interview contents (Table 5).

**Table 5:** Overview of the 25 code summary statements.

**Thematic field**	**Observation—community**
*Thematic code category*	*Positive non-systematic casuistic observations*
Summary statement	Examples of positive experiences of good HSICN, clinical progress post-discharge, family engagement
*Thematic code category*	*Negative non-systematic casuistic observations*
Summary statement	Casuistic observation of death shortly after discharge from neurological early rehabilitation
*Thematic code category*	*Facilitating aspects*
Summary statement	HSICN teams with a structured and reflective approach to their work and a good working atmosphere
*Thematic code category*	*Barriers*
Code subcategory “Nurses”—summary statement	Professional qualification variable and partially insufficient for specific neurorehabilitation aspects; main task seen in nursing care and patient safety only, not also in neurorehabilitation; work organization without reference nursing; changing team members and responsibilities
Code subcategory “Therapists”—summary statement	Too few community-based therapists; too few speech therapists with specific expertise (especially TC management); unclear medico-legal situation (delegation of medical services regarding TC management)
Code subcategory “Physicians”—summary statement	Family physicians have insufficient expertise for specific neurorehabilitation aspects; specialist diagnostics and therapy cannot be guaranteed in the community
Code subcategory “Technical aids”—summary statement	Individual supply can frequently not or only with delay be realized (healthcare insurance)
Code subcategory “Medication”—summary statement	Problems with prescription, organization, and delivery
Code subcategory “Networking”—summary statement	Interprofessional exchange not feasible, not remunerated
**Thematic field**	**Observation—outreach intervention**
*Thematic code category*	*Intervention success*
Code subcategory “Healthcare”—summary statement	Observed clinical improvements following recommendations given by ROFTs and their implementation
Code subcategory “Networking”—summary statements	Network building success (appreciative communication, joint goal setting, professional exchange with HSICN and speech therapy) Information for relatives increases their satisfaction and reduces the burden on HSICN
*Thematic code category*	*Intervention failure*
Code subcategory “Healthcare”—summary statement	Recommendations not implementable in the community setting (medication prescription)
Code subcategory “Networking”—summary statement	Cooperation difficult (lack of acceptance of ROFT members as a team member by HSICN, lack of HSICN staff, insufficient availability of therapists)

**Table 5:** (Continued)

*Thematic code category*	*Aspects of patient folder*
Code subcategory “positive”—summary statement	Patient folder covers relevant clinical areas, is easy to complete, and is sufficiently individualized
Code subcategory “negative”—summary statements	Not required with verbal agreements; not digital (limited user-friendliness, accessibility) Insufficient contextual reference (expertise, implementation)
*Thematic code category*	*Interpersonal/emotional reaction*
Code subcategory “positive”—summary statement	Positive emotional experiences of ROFTs during follow-up visits (during contact with known previous patients, HSICN, relatives)
Code subcategory “negative”—summary statements	Stressful emotional experience of ROFTs due to the limitation of expertise among community healthcare professionals and when faced with supply limitations Anxiety among family members associated with/induced by ROFT recommendations
**Thematic field**	**Expectation—community**
*Thematic code category*	*Expectation reflection, healthcare*
Code subcategory “positive”—summary statements	Patients will be well cared for in HSICNs; neurorehabilitation centers do not need to worry about “giving up” on patients with discharge to the community Bigger potential for patients’ improvement with some HSICNs with very high levels of care, education for neurological patients, and networking with other healthcare professions; they will not need support from a neurorehabilitation center
Code subcategory “negative”—summary statements	Care in the HSICN serves primarily to maintain the medical condition stable and prevent deterioration and complications (not: reaching further rehabilitation goals) Lower potential for patients’ improvement with some HSICNs with a very low level of care, education for neurological patients, and networking with other healthcare professions; high need for support by the neurorehabilitation center cannot be met by the ROFT Shortage of therapists in the community and consequently expected undersupply of patients, with hardly any qualifications for TC care available in the community Patients might acquire complications when emergency care in a non-specialized acute care hospital will be necessary
**Thematic field**	**Expectation—outreach intervention**
*Thematic code category*	*Expectation reflection, clinical pathway/medical aspects*
Code subcategory “positive”—summary statement	Community physicians apply clinical reasoning (medication)
*Thematic code category*	*Expectation reflection, clinical pathway/medical aspects*
Code subcategory “negative”—summary statements	Expected clinical content limitations (no rehabilitative improvement goals and treatment standards established) Foreseen resource limitations (medical expertise; availability and qualification of therapists; unsatisfactory supply of aids) Medico-legal issues (limitations for delegation of medical services to other healthcare professionals; narrow limits of ROFT responsibility)
*Thematic code category*	*Expectation reflection, clinical pathway/networking*
Code subcategory “positive”—summary statements	Regular contact between ROFT and HSICN will support trusting cooperation (preferably verbal/personal) ROFTs can network with both community-based therapists and relatives
Code subcategory “negative”—summary statements	ROFT/HSICN collaboration will be complicated by differing goals (functional progress vs. medical stability only), limited willingness to work extra hours, competency issues, unclear decision-making authority, risk management Cooperation with doctors (professional uncertainties of non-specialist family physicians in charge), therapists (time resources), relatives (commitment, resilience) will be difficult
*Thematic code category*	*Understanding of ROFT role*
Code subcategory “Regional outpatient follow-up team (ROFT)”—summary statements	ROFTs consider it their task to provide info on the project, suggest solutions (care, esp. therapeutic; for problems that arise), and inform relatives. ROFTs need a high level of frustration tolerance. Disadvantages of intervening in a “running” care reality “from outside”; lack of established risk protection/management in the community for the implementation of recommendations issued by the ROFT
**Thematic field**	**Familiarity aspects—related to ROFT team member**
Summary statement	ROFTs show familiarity with impairments and activity limitations, their relevance for daily living, treatment goals and recommendations, individual training needs, risk management, and privacy/data protection

HSICN, home-based specialized intensive care nursing; ROFT, regional outpatient follow-up team.

These statements will be verbally summarized and illustrated with examples below.

Regarding the observed healthcare situation in the community, the teams identified numerous healthcare-related barrier observations, including a lack of qualified professionals and limited expertise in specific neurorehabilitation issues. They reported highly variable, often negative, clinical care experiences in this context. For example:

“So I often see in the field of HSICN that the main mandate to act is to ensure that the patient is SAFE, CLEAN, SATIATED […] But it’s not the case that these ADVANCED rehabilitative areas, if it’s not someone who really enjoys working with the person they are caring for, then it’s a case of service to rule.” (T)Example from Table 4a.

“I think I would add one more thing about therapists or therapies. Yes, one of the challenges is to find at all therapists who in particular can provide care in rural areas, let alone suitable therapists who have already worked with neurorehabilitation. Perhaps also, especially in the case of speech and language therapists, we see that there is simply one practice, for example, where one speech and language therapist can unblock and as soon as she is absent, other things happen in therapy. Which does not necessarily have to be wrong, but certain processes or certain goals simply cannot be pursued for weeks because there simply is not the right staff on site or, for example, the HSICN leaves it entirely to the speech and language therapists or vice versa.” (T)Example from Table 4a.

“With patient conferences, the way we can organize it here in the hospital, that’s not possible. Besides, our therapists here in the hospital, if we schedule the patient for a conference now, they get paid. The therapist in the community either takes the patient hour, which would be quite dramatic, or he takes it from his free time, but why?” (P)Example from Table 4a.

Consequently, the teams expressed predominantly negative expectation reflections regarding the care situation for the specific patient group in the community.

“It’s easy to see what a speech and language therapist is, what a speech and language therapist is in that sense, or simply this swallowing therapist. Or this qualification of respiratory therapist, which would be so important in every HSICN. To see that there are things, I do not want to say “that hurt.” But you think: “If all this were there now, you could really do more for the patient, you could achieve even more.” That’s the thing, yes. That’s this negative, not negative, simply the experience that we have had, that we would not have thought that we would encounter this problem.” (N)Example from Table 4c.

“The implementation of high-frequency therapeutic therapy, especially speech therapy or respiratory therapy /. Because it would actually be desirable for the patient to receive care and treatment daily, or at least realistically three to four times a week. But sometimes, as I said, it’s not even possible once a week.” (T)Example from Table 4c.

Regarding observations made in the context of their own intersectoral outreach activity (NFC), expressions mostly indicated intervention success.

“And what else we can do, and we have already done this, is to give our e-mail address to the speech and language therapists and say: “Okay, just write to us if you have any questions. Call us on this number if you have anything. If you do not know how to proceed or if there is a regression. Simply report any observations.” The HSICN does this anyway in the daily exchange, so that they can report back to us. And as I said, we also get the speech and language documentation. If we noticed something, we would report it back. And we try to support the speech and language therapists who are on site as much as possible and simply offer them a certain level of security. That they also have more confidence to take part in tracheostomy tube management and help drive it forward.” (T)Example from Table 4d.

Nevertheless, negative expectation reflections on the implementation of the CP prevailed in terms of both medical aspects and concerning networking, for example:

“Well, the therapists in the HSICN, of course they also make their documents. My experience with the nursing staff is that they are very careful. And I think the only way we could implement therapy suggestions better would be if we had more therapy hours and the nursing staff were better equipped or more staff were available. And this staff would also be better trained. They are all very good and they do everything they can. But if I was a nurse in the HSICN and I did not have a doctor at my side, I did not have a therapist at my side, I would be VERY careful.” (P)Example from Table 4d.

“With patient conferencing, the way we can organize it here in the clinic, that’s not possible. Besides, our therapists here in the clinic, if we schedule the patient for a conference now, they get paid. The outpatient therapist either takes the patient hour, which would be quite dramatic, or he takes it from his free time, but why?” (P)Example from Table 4d.

After extensive code generation of various aspects and coding, it was then possible to recognize that very similar aspects, related to the community-related healthcare situation and the conceptualization, contextualization, and implementation of the CP, were mentioned by all three teams independently. The three different regional locations of the teams did not influence the results. It can, therefore, be assumed that the content of the results was saturated by the three multiprofessional, independent ROFTs.

### Deducing a conceptual map based on the thematic analysis

**Figure 2:**
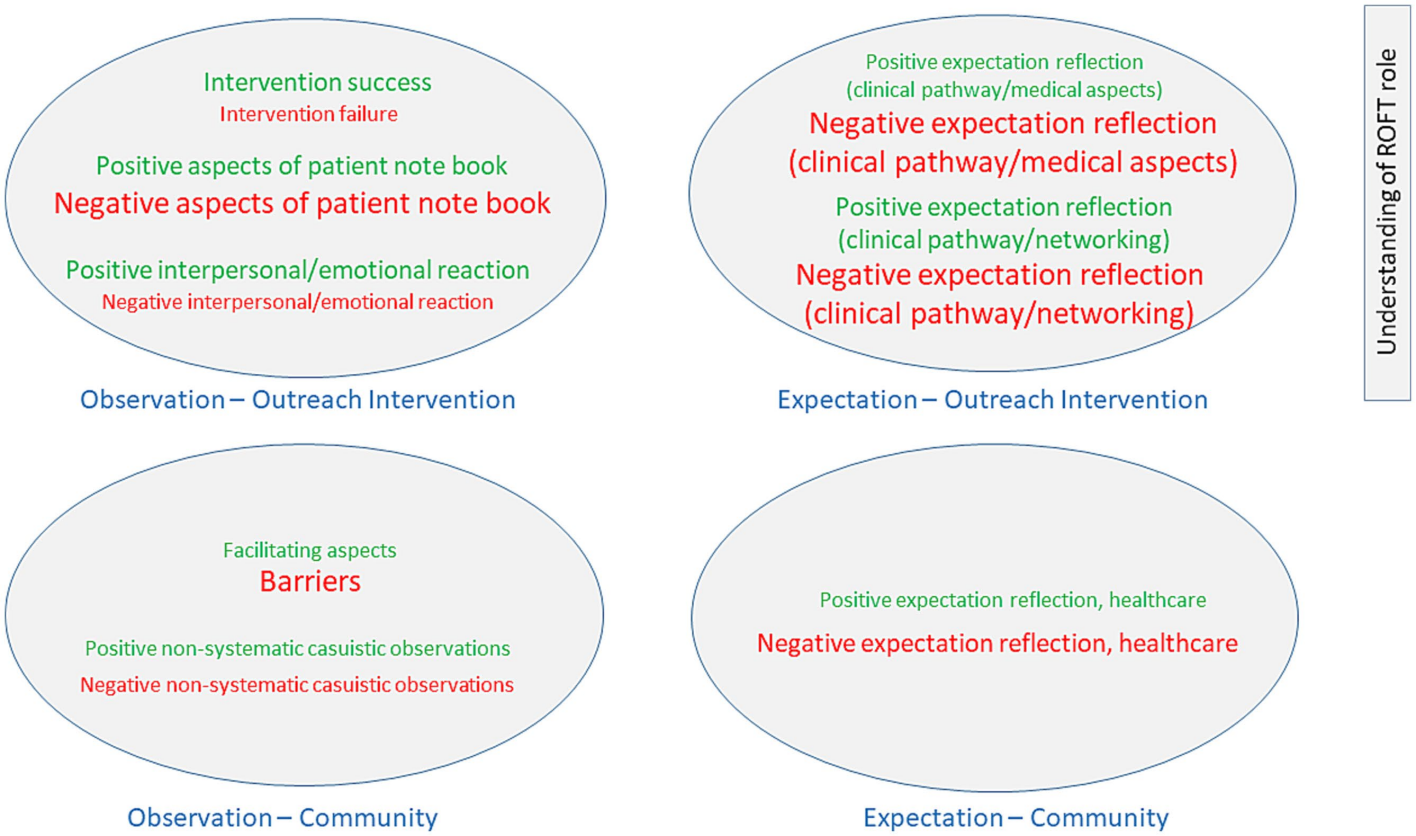
ROFT responses to observations and expectations regarding community healthcare, and, based on these, the observations and expectations related to outreach intervention. *Explanations:* The three font sizes illustrate the frequency with which the thematic category was mentioned, indicating the relevance of that thematic content, and are consistent with the qualitative thematic analysis. Font size 14 = 1–6 code mentions. Font size 17 = 10–19 code mentions. Font size 20 = 27–69 code mentions.

In the conceptual map of interview responses, the thematic topics emerging from the unique responses given by the ROFTs are graphically illustrated (Figure 2).

In the lower part of Figure 2, the responses regarding observations and expectations of the healthcare situation in the community for the clientele and service area are shown, and in the upper part of the figure, the responses regarding observations and expectations of the project’s outreach intervention (NFC) are presented. In addition, the ROFTs commented on their roles within the healthcare context in the fifth segmented thematic field.

For community healthcare, the ROFTs’ observations of barriers predominated, leading to predominantly negative expectations regarding adequate and further recovery-promoting healthcare of the specific clientele in the community. However, this was mirrored by (while less frequently mentioned) positive healthcare observations and consequently expectations, indicating considerable variability of healthcare provision in the community.

With regard to the outreach intervention (NFC), the ROFTs more frequently reported their intervention success and positive reactions to it from healthcare professionals in the community, while observations regarding the applicability of the patient folder (documentation aid for the individualized implementation of the CP) were more frequently negative due to the perceived limited willingness to use it in addition to the available (mono-professional) documentation systems among the various community-based professions. The observations of barriers further resulted in more negative expectations regarding both medical and networking aspects for the implementation of the clinical pathway, while positive expectations were also mentioned, again indicating a variability of healthcare situations experienced across individuals.

## Discussion

### Clinical relevance of the research topic

With advances in intensive care, more critically ill persons survive, but suffer from severe neurological deficits caused by either a primary neurocondition (such as stroke or traumatic brain injury) or a post-intensive care syndrome (PICS) (11). Although neurological early rehabilitation (NER) is frequently successful and associated with the possibility of returning home or to usual residential care, a certain proportion of those who are neurologically severely affected still have a need for intensive nursing care when discharged from the NER, either because of an ongoing need for mechanical ventilation, or severe dysphagia—frequently associated with severe paresis and/or disorders of consciousness—and a need for blocked tracheal cannulas (TCs) and permanent qualified medical supervision (3). In this situation, home-based specialized intensive care nursing (HSICN) and family physicians in charge provide the primary healthcare needed in the community. However, specific neurorehabilitation needs are not covered as this expertise is only available in regional NER centers, but not in the community. The project OptiNIV was set forth to develop, implement, and evaluate a “new form of care” (NFC) where healthcare professionals in any community of Bavaria (Federal State of Germany) providing such care (i.e., HSICN) are supported by multiprofessional outreach teams based in one of three participating regional NER centers (5).

### Elements and organization of the clinical pathway

An evidence- and guideline-based clinical pathway (CP) had been developed for the intersectoral co-treatment of severely affected neurological patients requiring intensive nursing care in the community after discharge from NER.

Its concepts and work aids were used by the ROFTs on a patient-centered basis, and an individualized patient folder was generated to support its implementation. The patient folder was designed to be accessible to all professions in the patients’ homes and to serve as a tool for mutual interprofessional information exchange.

Overall, the CP concept and working aids guide a person-centered, multidisciplinary healthcare approach with a rehabilitative orientation. Its implementation seeks to support intersectoral and multiprofessional cooperation between healthcare professionals from inpatient neurorehabilitation centers and those in charge in the community.

With this qualitative research, the CP’s conceptualization and associated documentation aids were reviewed after their implementation in practice.

### Strengths and weaknesses of the CP during implementation

During the interviews, the ROFTs did not question the concept of the CP. The CP was described as coherent and practical. Importantly, when reporting on the effects of their activity (NFC), they more frequently reported an intervention success. Personal and emotional reactions of community healthcare workers to their expert advice were also more often reported as positive.

However, they considered the existence of a team of qualified medical professionals in nursing care and therapy with qualifications and experience in neurorehabilitation in the community as a basic prerequisite for implementing CP. Yet, they noted that such a team was often missing in the community. Accordingly, they judged the basis for cross-sectoral cooperation between NER experts and community healthcare workers to be variable and frequently weak. As a result, they found it difficult to develop appropriate and individually applicable recommendations for the community care situation and thereby to positively shape the individual’s clinical progress. To them, the allocation of medical competencies—as another prerequisite for medical activities—also frequently remained unclear. While a highly vulnerable group of patients was served, they observed that organizing emergency handling was no different from the general population, which was not considered optimal.

The picture presented was, however, not uniform. On the contrary, the individuals’ healthcare situation regarding qualification, expertise, and type of healthcare professionals involved was reported as highly variable according to the interview partners’ perceptions, with high standards in a few well-organized situations.

Another topic was the proposed documentation using the patient folder. As HSICN and the various therapy professions individually used separate documentation systems, it was frequently difficult to implement this uniform, cross-professional joint documentation. At times, the ROFTs had to merge the information themselves from the various documentation systems, which meant a considerable amount of extra work to generate and update the patient folders.

Overall, the interviewed ROFTs’ expectation that the CP could be adequately implemented was more frequently negative. This position could not be related to a lack of the ROFTs’ reflection of their professional role or familiarity with issues at stake, as indicated by corresponding interview responses. Moreover, when prerequisites were met on an individual basis, their expectations were also positive.

Overall, a picture emerged indicating the potential of the CP to support healthcare of a clientele with very specific healthcare needs based on a hybrid collaborative model of NER center-based experts supporting community-based healthcare workers, both with regard to its acceptability and effectiveness (as subjectively perceived by ROFT members). This potential was counterbalanced by various contextual barriers and facilitators that needed to be addressed.

### National and international transferability of CP

This research nevertheless supports the CP conceptually, highlighting its relevance to other similar healthcare situations as a starting point for implementation.

For the development of the CP and user-friendly practical implementation aids in checklist format, international evidence-based guidelines were used on the topics of neurological rehabilitation in coma and severe impairment of consciousness (12), non-invasive and invasive ventilation as therapy for chronic respiratory insufficiency (13), prolonged ventilation weaning in neurological-neurosurgical early rehabilitation (14), positioning therapy and early mobilization for the prophylaxis or therapy of pulmonary dysfunction (15), and neurogenic dysphagia (16). Despite the developmental universality of the CP, its applicability depends largely on contextualized implementation based on specific efforts such as the identification of regional facilitators and barriers, as well as the development of contextualized implementation strategies and their monitoring.

With regard to the CP’s implementation, the qualitative research results further provided important insights. Long-term care for neurologically severely affected patients requires community-based multidisciplinary rehabilitation teams that can adapt evidence-based recommendations to the local context of the individual patient. These teams can benefit from accompanying training, support, and supervision by specialists from neurorehabilitation centers, who transfer specific tasks in care to the community representatives based on diagnostics, assessment, experience, and knowledge, including guidelines and protocols (17).

It is important to actively involve all team members from the various community-based professions in strategically managing community-based rehabilitation to support patients’ goals. By organizing treatment together, everyone involved can continuously improve their planning and thought processes to benefit patient care (18). As early as 2010, the WHO (19) emphasized that successfully implementing community-based rehabilitation requires providing the necessary framework conditions. As an added and innovative feature of the healthcare and research project reported here, the specialists’ expertise from neurorehabilitation centers is made available for the care of individual patients in the community. Together, the main tasks of the professions are the provision of needs-based care adapted to the community setting, the health activation and empowerment of patients, and the promotion of community support networks (20). The organizational structures necessary for such endeavors are time for interdisciplinary exchange, presence of a moderator for interprofessional communication, patient-specific knowledge, an agenda for structured team discussions, and uniform terminology/language (21). Communication between professions is decisive for the success or failure of intersectoral cooperation and the achievement of the patient’s rehabilitative goals (22). The intersectoral work in a regional network model should be a key element of community-based rehabilitation (23).

The evidence- and guideline-based CP and its working aids can facilitate both a person-centered care with an ongoing neurorehabilitative perspective, interprofessional networking, information flow, and expert guidance. Its constituents support both the necessary organizational and medical aspects and their documentation.

### Limitations of the results

Methodologically, both a rigorous multi-step qualitative research process based on consensus of two or more researchers and the consistency of responses across teams add to the credibility and transferability of findings to other similar healthcare situations. The researchers themselves (from a different federal state) had the role of independent researchers with a comparable clinical background. Although the interview was structured to be informative for the research question, the qualitative analysis of responses was open and identified emerging content and themes based on the experience, insights, and reflections as expressed by the interview partners. Various limitations were nevertheless identified for this study.

Only three ROFTs participated in the NFC project and could hence be interviewed. However, as the three regionally different teams independently of each other expressed very similar aspects regarding the appropriateness of the CP’s conceptualization and the context-specific experiences made during its implementation, it can be assumed that the results are saturated.

The qualitative interviews were conducted with three teams from three neurorehabilitation centers covering the intersectoral service in one (the largest) federal state of Germany, i.e., Bavaria. Therefore, a regional bias cannot be ruled out while being representative for the covered region.

Furthermore, the research addressed only the ROFTs’ perspective, but not that of healthcare workers from the community or people with neurodisabilities in need for HSICN, restricting the scope of the results. Their perspectives will be the focus of future research to provide a more complete understanding of the healthcare situation and the appropriateness of the CP.

In addition, the severely affected patient group treated in this project is less common than major treatment indications in the community, such as hemiparesis after stroke; the healthcare situation and prerequisites for an intersectoral network for more frequent conditions might well be different.

Before the study project, the teams at the three rehabilitation centers had little experience with intersectoral work, and hence the task of intersectoral interprofessional communication and case management. Although a lack of such prior experience might have made the CP’s implementation more difficult for them, any facilitators and barriers for its implementation were likely to be observed by them, though. Finally, as this work is qualitative research, no claim is made as to the representativeness of the data, and no hypothesis testing was intended.

### Health policy and practical consequences

Through this qualitative research, healthcare policymakers are informed that a hybrid collaborative center- and community-based healthcare approach for a clientele with highly specialized healthcare needs can be a model to address specific needs and potentially promote healthcare in the community. Although subjective, the reported intervention successes and the perceived acceptance by community healthcare providers support its usefulness. Healthcare policymakers could consider such a model, as it has the potential to foster specialized care in the community and reduce the load of in-hospital consultation and treatment.

For such collaborations of extended teams (based in hospitals and the community), the research results point to the importance of defining the roles and responsibilities of the multidisciplinary team members (24).

The identification of barriers and facilitators helped to identify a range of effective measures that might improve the contextual conditions for the successful implementation of the CP.

One approach could be targeted qualification measures for community-based healthcare workers to improve their knowledge and skills for selected critical care topics. Curricula for certificate education on clinical topics of high relevance and little prevailing expertise in the community, such as TC management, can be developed to ensure high-quality patient care. These can be used to provide in-depth training for therapists with certificates as proof of the key qualification acquired, enabling the allocation of medical activities covered by their competencies (25). Another example would be a certificate course for therapists and nursing staff to become respiratory therapists (26).

Furthermore, the insufficient density of therapists, especially in rural areas, could be addressed through telemedical applications (26). Based on telemedical therapy applications supervised by NER-based therapists, higher therapy frequencies could be made possible, as the effectiveness of telemedical applications can be comparable to that of face-to-face therapies. Telerehabilitation applications could further support interaction among medical professionals, e.g., for multidisciplinary case conferences without a need to meet physically, or with relatives to address their information needs effectively.

The establishment of trans-sectoral purpose-built digital patient folders is another option. In the SwissNeuroRehab project, conventional neurorehabilitation approaches are combined with new digital technologies to document patients’ personalized medical information and care across sectors from the university hospital to the community therapist, supporting a distributed online availability of person-centered information and exchange among professionals with a continuum of care perspective for neurorehabilitation (27).

Alternatively, a case manager can be employed to act as an organizational link between center-based healthcare and the community and among the otherwise non-linked healthcare workers in the community. In the STROKE OWL project, case managers were deployed to coordinate therapies, assist with applications, advise and motivate patients and their relatives, and monitor their medication and lifestyle (28).

A further option is the establishment of outpatient medical treatment centers at hospitals, e.g., as exemplified for adults with intellectual disabilities or severe multiple disabilities (MZEB), where patients from the community have the opportunity to attend outpatient special consultations in expert centers and receive treatment there (29).

## Conclusion

The qualitative research contributed to our knowledge regarding the CP’s practicality and appropriateness. Overall, in the interviews, the teams described the healthcare situation experienced in the community more extensively rather than discussing the concept and design of the CP itself. While applying the CP, the teams noted acceptance by community healthcare workers and intervention successes. Thus, no significant need to modify the concept of the CP emerged from the qualitative research.

Qualification for neurorehabilitation was, however, reported to be heterogeneous and limited in the community, more frequently making it difficult to implement the CP and to make good use of the associated documentation aids in the community.

Based on standardized terminology and professional qualifications in neurorehabilitation (21), intersectoral work in a regional network model can be a key element of community-based rehabilitation (17, 23). To develop guideline- and evidence-based CPs in such projects, implementation questions need to be addressed comprehensively and with the participation of all stakeholders involved (9). As illustrated by the qualitative research results presented, this includes both the analysis and targeting of contextual medical and interdisciplinary work barriers and the use of facilitators to generate the intended healthcare benefits. The provision of expert knowledge within intersectoral work is an essential, but not sufficient, prerequisite in a regional network model to promote a “continuum of care” approach for neurorehabilitation.

The important implications of the findings from this qualitative research are of interest to various stakeholders, including healthcare professionals in NER facilities and the community, persons with severe neurodisabilities, healthcare insurers, and politicians alike.

The use of hybrid care models that make specialist expertise from hospitals accessible to community healthcare workers and their patients offers a great potential for the optimal use of existing resources to support a continuum of care for patient populations with highly specialized healthcare needs that cannot be comprehensively met by the community healthcare system alone.

Barriers for implementation identified can be addressed by targeted solutions such as comprehensive training courses for community-based healthcare professionals supporting the acquisition of key qualifications, telemedicine applications (for interprofessional exchange and therapy), or digital medical records to be shared and used along the continuum of care from the hospital to the community.

After the successful conceptualization and contextualization of the CP with the help of the ROFTs, we aim to further evaluate the CP by interviewing healthcare professionals from the community to integrate their perspective.

## Clinical message

- Intersectoral work in a regional network model linking center-based neurorehabilitation expertise to community healthcare can be a key element of community-based rehabilitation, especially for less common complex neurological conditions.
- Guideline- and evidence-based CPs help to structure and facilitate intersectoral healthcare.
- CPs for intersectoral work need to be contextualized and implemented collaboratively, involving all stakeholders.
- Standardized terminology and professional qualifications in neurorehabilitation are essential for successful intersectoral cooperation.
- Interdisciplinary communication is a key element for teamwork, for community-based rehabilitation and intersectoral healthcare pathways.
- Implementation of CPs for intersectoral work needs to be coupled with the analysis and targeting of contextual medical and interdisciplinary work barriers and facilitators to generate the intended healthcare benefits.

## Data Availability

The original contributions presented in the study are included in the article/Supplementary material, further inquiries can be directed to the corresponding author.
