# Supplementary material for: Post-discharge “continuum of care” clinical pathway for persons with severe neurodisabilities—qualitative research to assess its concept and practicality after implementation
Source: Front Neurol. 2025 Sep 29;16:1552692. doi: 10.3389/fneur.2025.1552692 (PMC12517222; doi:10.3389/fneur.2025.1552692)
Supplement: Supplementary file 1 [file Table_1.DOCX]

**Supplementary table 1 (A)**. Overview of sample statements from all code- categories [original transcript in German]

| **Themenfeld** | **Beobachtungen – Ohne Interventionseinfluss** |
| --- | --- |
| *Thematische Code-Kategorie* | *Positive nicht-systematische kasuistische Beobachtungen* |
| Code-Subkategorie  „Versorgungssituation“ -  Aussagen-Beispiel | „Wir haben das eine Mal in ergotherapeutischer Sicht kurz gesehen, dass da Materialien von der Tochter und den Enkelkindern hergestellt wurden für feinmotorische Arbeiten.“ (T) |
| *Thematische Code-Kategorie* | *Negative nicht-systematische kasuistische Beobachtungen* |
| Code-Subkategorie  „Klinischer Verlauf“-  Aussagen-Beispiel | „Und was ich sagen muss, wo ich auch extrem überrascht war, wir hatten ja die zwei Patienten, die so schnell gestorben sind /. Ich habe nicht damit gerechnet, dass die ersten zwei Wochen so kritisch sind. Ich dachte, dieser Zeitpunkt der kritischen Phase, der kommt später. Aber dass der so früh, so schnell ist, damit habe ich nicht gerechnet.“ (A) |
| *Thematische Code-Kategorie* | *Fazilitatoren* |
| Code-Subkategorie  „Interprofessionelle Zusammenarbeit“-  Aussagen-Beispiel | „Man merkt auch schon innerhalb des Teams werden Die Kameraden, Die saßen dann gemeinsam beim Kaffee, haben sich ausgetauscht.“ (P) |
| *Thematische Code-Kategorie* | *Barrieren* |
| Code-Subkategorie  „AIP“-  Aussagen-Beispiele | „Was können die? Was wollen die denn? Dann bekommt man Antworten: „Ja, ich bin jetzt zwanzig Jahre schon in dem Beruf“. Man merkt aber, dass da seit zwanzig Jahren hat sich da auch nichts weiterentwickelt. Das ist, sind schon ganz verschiedene Aspekte.“ (P)  „Aber es gibt ein Thema, die Transkranielle Gleichstromstimulation […], die sicher, die ja in mancher Neurorehabilitation noch nicht mal durchführbar ist, wo man sicherlich in der Außerklinik nicht dran denken kann. Weil es weder den Facharzt gibt, der das supervidieren könnte, noch einen Therapeuten oder Pflegenden, der das anbringen könnte.“ (T)  „Also ich sehe im Bereich der AIP häufig, da ist der Haupthandlungsauftrag, der Patient muss SICHER, SAUBER, SATT sein […] Aber es ist nicht so, dass diese ERWEITERTEN rehabilitativen Bereiche, wenn es nicht jemand ist, der sich wirklich gerne mit der Person, die er pflegt, beschäftigt, ist erstmal Dienst nach Vorschrift getan.“ (T)  „Weil wir dadurch, dass natürlich ein Schichtdienst vorliegt, mit verschiedenen Personen, die auch innerhalb wechseln, ist es sehr schwierig, da eine Konstanz abzubilden.“ (T) |
| Code-Subkategorie  „Therapeut*innen“-  Aussagen-Beispiele | „Ich glaube, ein was würde ich noch zu den Therapeuten oder Therapien anfügen. Ja also, eine Herausforderung ist schon auch, Therapeuten überhaupt zu finden, die gerade vor allem auch ländliche Regionen versorgen geschweige denn auch geeignete Therapeuten, die eben schon mal was mit Neuroreha am Hut hatten. Vielleicht auch, also gerade bei den Logopäden sehen wir, dass es einfach zum Beispiel eine Praxis gibt, da kann halt eine Logopädin entblocken und sobald die ausfällt, passieren halt andere Dinge in der Therapie. Was nicht unbedingt falsch sein muss, aber einfach gewisse Prozesse oder gewisse Ziele einfach dann für Wochen nicht verfolgt werden können, weil einfach nicht das geeignete Personal vor Ort ist oder eben zum Beispiel die AIP das komplett den Logopäden überlässt oder andersrum.“ (T) |

*Abkürzungen.* AIP: Außerklinische Intensivpflegeteams; T: Therapeut (Ergotherapie/Logopädie); P: Pflegekraft und Studienassistenz; A: Arzt.

**Supplementary table 1 (B)**. Overview of sample statements from all code- categories [original transcript in German]

| *Thematische Code-Kategorie* | *Barrieren (Forstsetzung)* |
| --- | --- |
| Code-Subkategorie  „Therapeut*innen“-  Aussagen-Beispiele  (Forstsetzung) | „Und auch, was ich mitbekommen habe, ist, dass das Trachealkanülen-Management von Klinik zu Klinik unterschiedlich gehandhabt wird. Es muss eine Delegation vom Arzt auf den Logopäden erfolgen und wenn es die Klinik nicht macht, dann darf der Logopäde das gar nicht machen. Also das, nur weil man Logopäde ist, heißt das nicht, dass man automatisch die Befugnis hat, das Trachealkanülen-Management durchzuführen.“ (A) |
| Code-Subkategorie  „Ärzte“-  Aussagen-Beispiele | „Und vor allem, weil die keine Möglichkeit haben, bei den niedergelassenen Ärzten Hilfe zu suchen, also deswegen. Weil die niedergelassenen Ärzte oft auch gar nicht zurechtkommen mit den Patienten, mit dem klinischen Bild von diesem Patienten, die wir entlassen in so einem schweren Zustand.“ (A)  „Der HA an sich, das ist noch eine Generation, die sich schwer tut mit solchen Patienten.“ (P) |
| Code-Subkategorie  „Hilfsmittel“-  Aussagen-Beispiele | „Wir merken, dass die Patienten häufig sehr spät erst die Hilfsmittel bekommen. Die oft abgelehnt, auch oft der Stand oft gar nicht klar ist in der WG oder auch nur schwer zu erfragen ist.“ (T)  „Eine große Sache, an der wir regelmäßig hängen und auch jetzt versuchen, einen Fuß in die Tür zu bekommen, ist die Hilfsmittelversorgung. Wir merken, dass die Patienten häufig sehr spät erst die Hilfsmittel bekommen. Die oft abgelehnt, auch oft der Stand oft gar nicht klar ist in der WG oder auch nur schwer zu erfragen ist: „Wie sieht es denn nun aus mit dem Lifter? Ist der jetzt genehmigt? Ist der überhaupt, wurde der schon wieder abgelehnt? Wer kümmert sich denn da gerade drum?““ (T)  „Trotz, dass wir halt eine lange Vorlaufzeit von drei bis vier Wochen eigentlich schon hier in der Klinik haben. Und dass man Hilfsmittel, oder man da eigentlich alles, was nötig ist, schon in die Wege leitet. Das nichts destotrotz dann am Ende vieles nicht vor Ort ist.“ (A) |
| Code-Subkategorie  „Medikamente“-  Aussagen-Beispiel | „Oder eben Medikamente, die im Kliniksetting regulär verwendet werden, werden im ambulanten Bereich gestrichen, weil sie selbst zu zahlen sind.“ (P) |
| Code-Subkategorie  „Interprofessionelle Zusammenarbeit“-  Aussagen-Beispiel | „Austausch mit dem Therapeuten, wenn er jetzt nicht gerade zufällig davor die Stunde da ist, funktioniert nicht. Natürlich, der Austausch, die machen ihre eigene Dokumentation in der Patientenkurve in der AIP.“ (A)  „Mit Patientenkonferenz, wie wir das hier in der Klinik gestalten können, das ist nicht möglich. Außerdem, unsere Therapeuten hier in der Klinik, wenn wir den Patienten jetzt zu einer Konferenz ausplanen, der wird ja bezahlt. Der Therapeut ambulant, entweder nimmt er die Patienten- Stunde her, was ganz dramatisch wäre, oder er nimmt es von seiner Freizeit, aber warum.“ (A) |

*Abkürzungen.* AIP: Außerklinische Intensivpflegeteams; HA: Hausarzt; T: Therapeut (Ergotherapie/Logopädie); P: Pflegekraft und Studienassistenz; A: Arzt.

**Supplementary table 1 (C)**. Overview of sample statements from all code- categories [original transcript in German]

| **Themenfeld** | **Beobachtungen – Mit Interventionseinfluss** |
| --- | --- |
| *Thematische Code-Kategorie* | *Interventionserfolgserfahrung* |
| Code- Subkategorie  „Versorgungssituation“  Aussagen-Beispiele | „Also einen Patienten haben wir ja wirklich dabei, der macht Fortschritte über Fortschritte, der einfach im Klinikbereich an der Wand war […] der ist stagniert, ist nichts mehr weiter gegangen. Und jetzt in der AIP macht der Fortschritte.“ (P)  „Bei einem Patienten die, die ACV eingeführt, also diese Above Cuff Vocalisation, über die subglottische Absaugung. Ja, das war zum einen sehr sehr schön zu sehen, weil das Team sich dafür interessiert hat und auch diese Möglichkeit der eigentlich klinischen Behandlungsweisen für sich noch gar nicht so /. Also sie kannten das, die Logopädin, die Ergotherapeutin, das AIP- Team waren da und der Atmungstherapeut der WG. Die haben das sehr gut aufgenommen und waren verblüfft, dass der Patient sprechen kann, der bis zu dem Zeitpunkt noch nie gesprochen hatte.“ (T) |
| Code- Subkategorie  „Interprofessionelle Zusammenarbeit“  Aussagen-Beispiel | „Und ich muss ehrlich sagen, ich hatte auch den Eindruck, dass sowieso das Gespräch mit dem Angehörigen dazu geführt hat, wir auch wieder mehr Beziehungen mit der WG hatten. Dass die das auch so zur Entlastung empfunden haben, dass wir das investiert haben. Und da ist auch wieder die Zusammenarbeit oder so gewachsen ist.“ (A) |
| *Thematische Code-Kategorie* | *Interventionsmisserfolgserfahrung* |
| Code- Subkategorie  „Versorgungssituation“  Aussagen-Beispiel | „Oder eben so spezielle Sachen zur Sekret- Reduzierung. Dass die, wenn es als Tropfen, wird es, bei mir war es bei Gastrozepin, als Tropfen darf man es haben, aber wenn es IV ist, wird es nicht verordnet. Also es gibt, die sind so viel an die Wand gedrückt, die Wohngruppen. Also auch mit unseren Empfehlungen, das fand ich negativ beeindruckend.“ (P) |
| Code- Subkategorie  „Interprofessionelle Zusammenarbeit“  Aussagen-Beispiel | „Ja, kannte den Patienten nicht und genau so hat sich die Situation dann auch entwickelt. Da war es für uns nicht möglich, ins Team reinzukommen. Es war einfach kein Interesse da, genau, von keiner Profession. Weder von den Therapeuten, noch von der Pflege, noch vom Arzt war ein Interesse daran, dass wir quasi als Teammitglied auftreten durften. Da hatte man dann schon eher das Gefühl, dass es darum geht, wie „P“ es sagte, eher ein eingeschworener Kreis zu bleiben, warum auch immer.“ (T) |
| *Thematische Code-Kategorie* | *Aspekte Patientenhefter* |
| Code- Subkategorie  „Positiv“  Aussagen-Beispiele | „Also als Beispiel, der klinische Pfad an sich ist logisch.“ (T)  „Also grundsätzlich, das hat ein Konzept, das System.“ (A)  „Es sind trotzdem wichtige Punkte und gute Punkte, die man, wenn man das Blatt vorliegen hat, auch gut abarbeiten kann. Das ist dann auch schneller formuliert, mit einem Kreuz gesetzt, als das Ganze auszuformulieren.“ (T) |
| Code- Subkategorie  „Negativ“  Aussagen-Beispiele | „Also ich finde, man denkt auch oft so an die Frage, inwiefern jetzt eine händische Papierdokumentation, also ob nicht das langfristige Ziel natürlich wäre auch, ob so ein Austausch nicht auch digital funktionieren kann. Das eben alle Partner das THEORETISCH von ihrem Therapieschreibtisch, wo sie sowieso aus der Praxis heraus, dokumentieren und die Informationen senden können.“ (T) |

*Abkürzungen.* AIP: Außerklinische Intensivpflegeteams; T: Therapeut (Ergotherapie/Logopädie); P: Pflegekraft und Studienassistenz; A: Arzt.

**Supplementary table 1 (D)**. Overview of sample statements from all code- categories [original transcript in German]

| *Thematische Code-Kategorie* | *Aspekte Patientenhefter (Fortsetzung)* |
| --- | --- |
| Code- Subkategorie  „Negativ“  Aussagen-Beispiele  (Fortsetzung) | „Wir haben eigentlich eher im persönlichen Austausch entweder vor Ort oder telefonisch die beste Erfahrung gemacht. Das […] weißt du nicht, wann wird es gelesen, durch wen, was findet der denn davon. Wer nimmt es weiter auf.“ (A)  „Und ich glaube, in Bezug auf die Therapiedokumentation nochmal /. Wir sehen, dass es häufig in den AIPs selber eine Möglichkeit gibt, Therapien zu dokumentieren. Je nach Standort, je nach Therapeutenkonstellation wurde es dann benutzt oder auch nicht. Ich habe mich mit mehreren Logopädinnen auch schon unterhalten, da ist oft die Rückmeldung: „Ich muss ja sowieso für mich in der Praxis dokumentieren. Und dann muss ich in der AIP selbst und zu Hause sozusagen nochmal dokumentieren“. Und dass das dann übereinstimmt, da die gleichen Informationen drinstehen und das Ganze funktioniert, das scheint für viele halt einfach ein hoher Aufwand zu sein. Weil es einfach nicht so einfach eins zu eins übertragbar ist, wie ich schicke mal per E-Mail die Dokumentation von heute rum, das geht ja nicht einfach. Und, also das ist so eine Sache, die auch die Therapeutinnen angeben, die einfach jetzt auch, wir hatten den klinischen Pfad ja auch einen Teil mit der Therapiedokumentation noch mit drin. Das wäre dann ja das dritte Dokument, in das man etwas reinschreiben könnte.“ (T)  „Vielleicht kann man zu dem Thema noch sagen, dass wir uns auch mit dem Thema Datenschutz diesbezüglich noch befassen wollten.“ (T)  „Und auch, weil die Ressourcen knapp sind, finde ich es vor allem ein sehr hilfreiches Tool für uns und finde es eigentlich, würde ich präferieren, es eher bei uns zu pflegen und haben. Und dann denen nur das dann nur zusammengefasst zu geben, was für die wirklich relevant ist beziehungsweise was vor Ort fehlt vielleicht. […] Um einen da reinzulesen und was dann wirklich die Empfehlung ist, die da rauskommt, das ist oft nur ein Satz oder so, was wir denen wirklich empfehlen würden. Und dann würde ich auch nur den Satz kommunizieren und vielleicht auch eher dann in einem Telefongespräch, wo man sich dann auch gleich darüber austauschen kann. Ich glaube, dann ist es auch einfach ein fruchtbarerer Boden, worauf es fällt.“ (A) |
| *Thematische Code-Kategorie* | *Interpersonelle/ emotionale Reaktion* |
| Code- Subkategorie  „Positiv“  Aussagen-Beispiele | „Und wie sicher sich Angehörige fühlen, wenn sie wissen, sie werden von diesen Menschen mit weiter begleitet.“ (P)  „Die Erfahrung haben wir jetzt gerade noch nicht so gemacht. Was ist, dass wir am Anfang […] von den Patienten, die aus dem eigenen Stall kommen, da werden wir immer wieder so /, richten Grüße aus, freuen sich, dass wir vorbeikommen, oder man grüßt zurück.“ (P)  „Ja, das war zum einen sehr sehr schön zu sehen, weil das Team sich dafür interessiert hat.“ (T) |
| Code- Subkategorie  „Negativ“  Aussagen-Beispiele | „Und dann immer so im Hinterkopf, dieses Dagegenhalten /. Für diesen Platz wird ja unfassbar viel Geld gezahlt im Monat. Und dann sind das solche […] Kleinbeträge […], dass es an diesen Pfennigbeträgen scheitert.“ (P) |

*Abkürzungen.* AIP: Außerklinische Intensivpflegeteams; T: Therapeut (Ergotherapie/Logopädie); P: Pflegekraft und Studienassistenz; A: Arzt.

**Supplementary table 1 (E)**. Overview of sample statements from all code- categories [original transcript in German]

| *Thematische Code-Kategorie* | *Interpersonelle/ emotionale Reaktion (Fortsetzung)* |
| --- | --- |
| Code- Subkategorie  „Negativ“  Aussagen-Beispiele  (Fortsetzung) | „Also wir haben, als Beispiel, einen Patienten momentan in der Häuslichkeit, wo unglaublich große Ängstlichkeit bei den Angehörigen vorherrscht und jeder Schritt des AIP- Teams, egal ob er gut oder schlecht ist, quasi mit Argusaugen betrachtet wird. Am liebsten sollte jeder Schritt von uns gestempelt werden als „gut“.“ (T) |
| **Themenfeld** | **Erwartungen – Ohne Interventionseinfluss** |
| *Thematische Code-Kategorie* | *Erwartungsreflexion, klinische Versorgungsbeobachtung* |
| Code- Subkategorie  „Positiv“  Aussagen-Beispiele | „Den AIPs das Vertrauen gibt, daran hakt viel. Man hat immer so das Gefühl gehabt, ich komme aus vierzig Jahren Intensivpflege, wenn man Patienten dann ins häusliche, WGs und so weiter, dass dann so ein Aufgeben stattfindet. Und das ist ein Umdenken und das hat auch bei mir stattgefunden jetzt. War früher mit Sicherheit auch mehr so, ist /. Da hat aber auch ein Umdenken bei mir stattgefunden, also die Patienten, die wir jetzt beobachtet haben, die sind sehr gut aufgehoben.“ (P)  „Und auf der anderen Seite hat man auch WGs, die wirklich schon auf einem sehr hohen Niveau sind, wo alles schon läuft, die eine Vernetzung untereinander haben, wo die wirklich zusammenarbeiten mit Therapeuten, mit Ärzten. Wo man merkt, da sind wir eigentlich quasi nur noch, überflüssig nicht, aber wir, da gibt es für uns nicht mehr viel zu tun, weil es wirklich gut läuft.“ (P) |
| Code- Subkategorie  „Negativ“  Aussagen-Beispiele | „(…) aber wo wir jetzt sind, in XYZ, da ist eigentlich, ja, nur noch Zustandserhaltung, keine Verschlechterung.“ (P)  „(…) und gehen zu anderen Teams weiter mit anderen Patienten, die schon entlassen wurden mit Dekubitus und die die ganze Zeit auf dem Rücken liegt und gar keine Möglichkeit hat zur Lagerung und so Hilfsmittel für Lagerung, es gibt keine, also es ist so sehr unterschiedlich.“ (A)  „Also so spezielle Sachen, das ist schon schwierig, das kennt da draußen noch keiner. Also die Patienten, die jetzt im minimalen Bewusstsein sind, da geht das über die Standardtherapie mit bewegen und der normalen Pflege eigentlich nicht hinaus, wenn man ehrlich ist, also die fallen subjektiv hinten runter. Auch wenn wir jetzt noch nicht so viele hatten.“ (T)  „Aber der Personalmangel, der ist im ländlichen Raum nochmal extremer.“ (A)  „Einfach zu sehen, was ist ein Sprachtherapeut, was ein Logopäde in dem Sinn oder einfach so dieser Schlucktherapeut. Oder auch diese Qualifikation des Atemtherapeuten, die so wichtig in jeder WG wäre. Zu sehen, dass es, dass es schon so Sachen gibt, ich will jetzt nicht sagen: „Die weh tun“. Aber man so denkt: „Wenn das jetzt alles da wäre, könnte man für den Patienten wirklich mehr, könnte man nochmal mehr erreichen“. Das ist das, ja. Das ist so dieses Negative, nicht negative, einfach die Erfahrung, die wir gemacht haben, die wir nicht gedacht hätten, dass man auf diese Problematik stößt.“ (P)  „Die Umsetzung der hochfrequenten therapeutischen Therapie, insbesondere Logopädie oder Atmungstherapie /. Weil, es wäre ja eigentlich wünschenswert, wenn der Patient täglich oder zumindest im realistischen Sinn drei bis viermal die Woche betreut, behandelt, wird. Aber es ist ja teilweise, wie gesagt, nicht mal ein Mal die Woche möglich.“ (T) |

*Abkürzungen.* AIP: Außerklinische Intensivpflegeteams; T: Therapeut (Ergotherapie/Logopädie); P: Pflegekraft und Studienassistenz; A: Arzt.

**Supplementary table 1 (F)**. Overview of sample statements from all code- categories [original transcript in German]

| *Thematische Code-Kategorie* | *Erwartungsreflexion, klinische Versorgungsbeobachtung (Fortsetzung)* |
| --- | --- |
| Code- Subkategorie  „Negativ“  Aussagen-Beispiele  (Fortsetzung) | „Ich glaube, was man da auch noch anfügen kann, ist, dass viele AIPs eben auch von sich aus sagen, dass eben, dass auch so eine Angst vor Komplikationen da ist. Auch, wenn sie sagen, die Patienten sind oft gefühlt bei ihnen selbst besser versorgt als wenn sie jemanden ins Akuthaus verlegen. Die ja auch nichts über den Fall wissen und dann nochmal ein neuer Behandler sind in diesem ganzen Feld. Die gar keinen Kontakt zu dem äußeren System haben, in dem der Patient sich befindet. Und da auch manchmal dann Sachen umgestellt werden und die Patienten kommen zurück und alles ist anders und alles ist neu. Und sie haben teilweise, oder ihnen kommt es so vor, als hätten sie da eine instabilere Problematik oder System als zuvor. Und dann erbricht er vielleicht nicht mehr aber dann sind halt andere Baustellen, weil vielleicht irgendwelche Medikamente weggefallen sind oder solche Dinge. Also auch das hören wir immer wieder.“ (T) |
| **Themenfeld** | **Erwartungen – Mit Interventionseinfluss** |
| *Thematische Code-Kategorie* | *Erwartungsreflexion, Umsetzung klinischer Pfad medizinisch-inhaltlich* |
| Code- Subkategorie  „Positiv“  Aussagen-Beispiel | „Ich denke, da ist es noch am Strukturiertesten, weil das Clinical Reasoning im medizinischen Dienst, aus meiner Sichtweise als Therapeut, am Etabliertesten ist, weil man ja einfach ganz klar sieht: „Okay ich gebe ein Medikament zum Beispiel und erhoffe mir diese Wirkung. Und wenn die nicht eintritt, dann werde ich es absetzen oder nochmal drüber nachdenken, ein anderes zu nehmen“.“ (T) |
| Code- Subkategorie  „Negativ“  Aussagen-Beispiele | „Also ich finde, das kommt drauf an. Das ist sehr unterschiedlich von WG zu WG. Ich glaube, wenn man das, ich glaube viele haben ein funktionierendes System, was für SIE funktioniert. Ich sag jetzt mal, das Minimum, was sie brauchen für das tägliche Arbeiten, funktioniert. Für diesen Ansatz, dass wir WIRKLICH dieses Potenzial vollkommen ausschöpfen wollen, ist es natürlich eine andere Frage, ob dafür das System dienlich ist.“ (T)  „Sicher bezüglich so Standards: wann darf entblockt werden? Mit welchen Kanülen, et cetera. Das sind halt oft auch Sachen, die nicht wirklich niedergeschrieben sind. Es gibt dann halt irgendwo eine Pflegeleitung oder irgendwen, der da in seinem Kopf ein Konzept hat.“ (A)  „Und das andere ist, da ist natürlich auch schon das Eine oder andere vorhanden. Und das Problem ist natürlich, Doppeldokumentation mag keiner. Aber ein zusätzliches Problem ist natürlich, dass die Ressourcen eh schon sehr begrenzt sind. Sicher, was die Therapeuten angeht und die Ärzte. Und dass die in der jetzigen Situation schon sehr wenig dokumentieren. Und das zum Beispiel eigentlich jede WG hat irgendein Dokumentationsblatt für die Therapeuten. Wenn die nicht genutzt werden wird es halt auch nichts bringen, wenn wir noch eine zweite Edition hinlegen.“ (A)  „Und das ist, das gestaltet sich auch dann schwierig zu, weil es ja auch innerhalb eines Teams gibt es ja ganz unterschiedliche, ich nenne es jetzt mal, Qualitätsstufen. Das nicht so ganz einfach immer auch herauszufinden, wo befindet sich das Team jetzt?“ (P) |

*Abkürzungen.* AIP: Außerklinische Intensivpflegeteams; T: Therapeut (Ergotherapie/Logopädie); P: Pflegekraft und Studienassistenz; A: Arzt.

**Supplementary table 1 (G)**. Overview of sample statements from all code- categories [original transcript in German]

| *Thematische Code-Kategorie* | *Erwartungsreflexion, Umsetzung klinischer Pfad medizinisch-inhaltlich (Fortsetzung)* |
| --- | --- |
| Code- Subkategorie  „Negativ“  Aussagen-Beispiele  (Fortsetzung) | „Der Patient wurde hospitalisiert aufgrund von einer Kanülen- Fehllage. Hatte mehrere Sättigungsabfälle, die wirklich bedrohlich hätten werden können. Also bald an der Schwelle zu Bedrohlichkeit, eine Intervention musste sein. Und wenn bei diesen Patienten immer wieder nachgefragt wird, warum er denn ein Pulsoxymeter braucht, obwohl wir im Dekanülierungsprozess in der Theorie sind. Gehört das meiner Meinung nach in den klinischen Pfad, dass das auch ein Problem ist. Weil, das behindert letztendlich alle Teile des klinischen Pfades, weil wir durch den Kostenträger letztendlich den Block erhalten. Den wir erstmal formell bearbeiten müssen, auch wenn, rein technisch, das Gerät schon da ist. Aber es ist, nimmt unglaublich viel Raum, auch bei der AIP, ein: „Ja wir haben da wieder einen Brief bekommen, was machen wir denn da jetzt? Helft ihr uns dabei“? Das ist nicht einfach. Das ist, vielleicht ist das irgendwie abbildbar. Weil das ja auch, wie gesagt, Prozesse beschreibt.“ (T)  „Also, die Therapeuten in den AIPs, die machen natürlich auch ihre Dokumente. Die, meine Erfahrung ist mit dem Pflegekräften, dass sie doch sehr vorsichtig sind. Und ich glaube, die einzige Methode, wie wir Therapie- Vorschläge besser umsetzen könnten, wäre, wenn wir mehr Therapiestunden hätten und das Pflegepersonal besser ausgestattet wäre beziehungsweise mehr Personal vorhanden wäre. Und dieses Personal auch besser ausgebildet wäre. Die sind schon alle sehr gut und die machen auch alles, was sie können. Aber, dass, also wenn ich Pflegekraft in der AIP wäre und ich hätte keinen Arzt an meiner Seite, ich hätte keinen Therapeuten an meiner Seite, ich wäre SEHR vorsichtig.“ (A)  „Ja, also die Pflegekräfte sind, wie gesagt, sehr zurückhaltend und das verstehe ich aber auch. Die können nicht die Verantwortung tragen, wir tragen die Verantwortung ja auch nur für unsere Empfehlung, aber für die Umsetzung ist natürlich dann die entsprechenden Pflegekraft oder der entsprechende HA zuständig. Und die warten aber auch immer, und sollen sie auch, auf die Rückmeldung des betreuenden HA.“ (A)  „Also ich denke, dass ein guter Austausch nur durch regelmäßigen Kontakt funktionieren kann und dass das Team überhaupt Vertrauen zu uns fasst. Das sie auch sehen, dass wir im Notfall wirklich für sie greifbar werden. Aber tatsächlich ist es so, wenn da ein Notfall in der AIP ist, wir sind nicht erreichbar, können wir gar nicht leisten, so eine rundum Notfallbetreuung. Von daher verstehe ich auch, dass die Teams bei zu weit gegriffenen Therapien auch zurückhaltend sind.“ (A)  „Ich denke, eine Sache, die große Angst macht, ist die Haftungssache. Wer, wer ist verantwortlich, wenn es schief geht? Wenn der Patient, im guten Fall, nur eine Pneumonie bekommt, im schlechtesten Fall stirbt? Ich könnte mir vorstellen, dass da sofort, wenn da jetzt der Logopäde das ambulant angeordnet hätte, dass dann sofort alle sagen: „Ja, der Logopäde ist schuld“. Und da möchte ich natürlich nicht in dessen Haut stecken. Es wäre gut, wenn man das wieder auf eine ärztliche Haftung abwälzen könnte. Das ist wahrscheinlich so, so eine Problemkonstellation wie bei den Hebammen, die selbständig draußen tätig sind.“ (A) |

*Abkürzungen.* AIP: Außerklinische Intensivpflegeteams; HA: Hausarzt; T: Therapeut (Ergotherapie/Logopädie); A: Arzt.

**Supplementary table 1 (H)**. Overview of sample statements from all code- categories [original transcript in German]

| *Thematische Code-Kategorie* | *Erwartungsreflexion, Umsetzung klinischer Pfad interprofessionelle Zusammenarbeit/Teambildung* |
| --- | --- |
| Code- Subkategorie  „Positiv“  Aussagen-Beispiele | „Und die Frage ist, glaube ich generell, wie funktioniert überhaupt dieser Wissenstransfer zwischen Therapeutinnen und der AIP. Und ich glaube, da passiert einfach sehr viel mündlich.“ (T)  „Also das habe ich eher als recht fruchtbar empfunden und denke auch, dass ein Austausch an verschiedenen Stellen Sinn macht, vielleicht nicht jede Woche. Aber ich glaube, dass so dieses, alle versucht man so ein bisschen in Kommunikation miteinander zu bringen, schon sinnvoll ist.“ (T)  „Also ich denke, dass ein guter Austausch nur durch regelmäßigen Kontakt funktionieren kann und dass das Team überhaupt Vertrauen zu uns fasst. Das sie auch sehen, dass wir im Notfall wirklich für sie greifbar werden.“ (A)  „Und was wir noch machen können, ist, das haben wir auch schon gemacht, die, unsere E-Mail- Adresse an die Logopäden rausgeben und sagen: „Okay, schreibt uns einfach, wenn ihr Fragen habt. Ruft uns an unter der und der Nummer, wenn irgendwas ist. Wenn ihr nicht wisst, wie ihr weiter machen sollt oder wenn es einen Rückschritt gibt. Alle Beobachtungen einfach mitteilen“. Macht die AIP sowieso im werktäglichen Austausch, dass die uns das dann auch rückmeldet. Und wie gesagt, die Logo- Dokumentationen kriegen wir auch. Wenn uns was auffallen würde, würden wir das wieder rückmelden. Und da versuchen, die Logopäden, die vor Ort sind, so gut wie möglich abzuholen und ihnen einfach eine gewisse Sicherheit bieten. Dass sie sich eben auch mehr trauen, am Trachealkanülen- Management teilzunehmen und das mit voranzutreiben.“ (T)  „Also wir wissen das ja von Anfang an überhaupt nicht, ob die die Patienten abdecken oder nicht. Wir rufen dann an und fragen: „Hey, hat jemand von euch Zeit? Habt ihr Kapazität? Wir bräuchten /“. Und dann sagt die Praxis: „Ja es geht oder nein, es geht eben nicht“. Und manchmal hat man ja so Wackelkandidaten oder so. Und dann kann man ja auch sagen: „Ja, es gibt Personal. Wir können ihnen, wir können sie unterstützen, wenn Rat gewollt ist“.“ (T)  „Weil, die Angehörigen und die AIPs haben wir ja schon vorher angefragt: „Hey, wir machen eine Studie, wollt ihr mitmachen“? Und die Angehörigen sind ja meistens sehr froh und sehr dankbar. Und ich hatte bisher noch nicht den Fall, dass die Angehörigen gesagt haben: „Nein, das Dokument darf jetzt nicht rausgegeben werden“.“ (A) |
| Code- Subkategorie  „Negativ“  Aussagen-Beispiele | „Und da ist dann oft auch nicht so klar, was darf zum Beispiel ein Therapeut selber entscheiden? Wo braucht er eine Einwilligung vom HA? Und das sind dann halt auch wieder so Sachen, wo Wochen ins Land gehen können und dann einfach wieder nix passiert. Und man wieder einfach dasteht, wo man angefangen hatte.“ (A)  „Weil wenn man das nicht kommuniziert, sagen schon die PDLs der Wohngemeinschaft: „Wenn meine Mädchen oder meine Leute, wird manchmal so genannt, wenn sie mehr machen müssen, dann machen wir es nicht“. Und die Schwelle ist unglaublich niedrig. Man denkt das gar nicht, aber die Schwelle, diese ABLEHN- SCHWELLE, ist unglaublich niedrig.“ (T) |

*Abkürzungen.* AIP: Außerklinische Intensivpflegeteams; HA: Hausarzt; T: Therapeut (Ergotherapie/Logopädie); A: Arzt.

**Supplementary table 1 (I)**. Overview of sample statements from all code- categories [original transcript in German]

| *Thematische Code-Kategorie* | *Erwartungsreflexion, Umsetzung klinischer Pfad interprofessionelle Zusammenarbeit/Teambildung (Fortsetzung)* |
| --- | --- |
| Code- Subkategorie  „Negativ“  Aussagen-Beispiele  (Fortsetzung) | „Ein Punkt, der mir gerade noch einfällt und wichtig ist, dass die halt auch ein anderes Ziel und ein anderes Denken haben. In der Reha sind wir halt: „Wie kann man vorwärtskommen und das Maximale erreichen“? Und die haben ein viel höheres Sicherheitsbedürfnis. Und das ist ja auch verständlich, weil, ich meine, wir haben unter Umständen ein Bronchoskop zick zack da oder was auch immer, ein Intensivteam. Das haben die da einfach nicht. Und das macht halt auch mit den Umsetzungen von unseren Empfehlungen /. Das es zum Teil dann auch einfach verständlich ist, dass wir da auf Widerstand stoßen.“ (A)  „Also wenn der HA, also wir haben das auch bei einem anderen Patienten, wenn der HA dann beim AFNT- Team anrufen muss, weil er, ganz natürlich, das ist ihm gar nicht vorzuwerfen, nicht weiß, wie er einen Hustenassistenten einzustellen hat, weil sich das AIP-Team den gewünscht hat. Ja aber komplett überfordert mit dieser Situation ist, dann die Angehörigen natürlich zurecht sagen: „Naja, aber warum wissen Sie das denn jetzt nicht? Sie haben das doch verschrieben“. „Ja, weil die Pfleger das wollten“. Und ja, da entstehen wirklich Vakuum- Zustände, die schwierig zu lösen sind dann auch.“ (T)  „Gleichzeitig habe ich schon den Eindruck, dass da schon ein Bedarf nach mehr multidisziplinären Austausch besteht. Aber das dann eher halt die Strukturen und auch die Zeit und natürlich am Ende die Finanzierung nicht so da ist, genau.“ (A)  „Und ich glaube auch, dass es für die Angehörigen /. Ich stelle mir das ein bisschen schwierig vor, weil ich als Angehöriger gebe meinen, wiederum, den Angehörigen in diese AIP, damit ich mich nicht selbst kümmern muss, weil ich mich nicht kümmern kann, weil ich vielleicht gar nicht emotional dazu in der Lage bin, weil ich mich vielleicht mit den Themen auch gar nicht auskenne. Wenn man, vielleicht sind dann diese Angehörigen dann erstmal froh, dass der Mensch gut behütet in dieser AIP ist. Und wenn man ihn dann wieder zurück mit ins Boot nimmt, um dieses ganze Klinische mit zu versorgen glaube ich, dass einfach viele Menschen abblocken. Weil sie sich, glaube ich, eher auf diesen emotionalen /: „es ist ja immer noch mein Partner, ist ja nicht ein Patient“ konzentrieren, denke ich.“ (T)  „Mit Patientenkonferenz, wie wir das hier in der Klinik gestalten können, das ist nicht möglich. Außerdem, unsere Therapeuten hier in der Klinik, wenn wir den Patienten jetzt zu einer Konferenz ausplanen, der wird ja bezahlt. Der Therapeut ambulant, entweder nimmt er die Patienten- Stunde her, was ganz dramatisch wäre, oder er nimmt es von seiner Freizeit, aber warum.“ (A) |
| *Thematische Code-Kategorie* | *Rollenverständnis der AFNT* |
| Code-Subkategorie  „Ambulantes Fachnachsorgeteam (AFNT)“-  Aussagen-Beispiele | „Und ein gewisses, es wird sicherlich auch von unserer Seite so sein, dass wir durch die Kommunikation des klinischen Pfades, durch die Routine, sicherlich dann uns auch mehr an die Situation der Außerklinik anpassen werden. Und dann vielleicht das Eine oder andere anders, besser, im Optimalfall kommunizieren können.“ (T) |

*Abkürzungen.* AIP: Außerklinische Intensivpflegeteams; HA: Hausarzt; AFNT: Ambulante Fachnachsorgeteams; T: Therapeut (Ergotherapie/Logopädie); A: Arzt.

**Supplementary table 1 (J)**. Overview of sample statements from all code- categories [original transcript in German]

| *Thematische Code-Kategorie* | *Rollenverständnis der AFNT (Fortsetzung)* |
| --- | --- |
| Code-Subkategorie  „Ambulantes Fachnachsorgeteam (AFNT)“-  Aussagen-Beispiele  (Fortsetzung) | „Deswegen solche Themen wie ein Entblocken oder Weaning-Sachen, was ja auch ein Teil vom Projekt ist, damit reinzugehen, einfach mit Vorsicht betrachtet werden aus der AIP. Hürden gibt es viele, glaube ich. Die Frage ist ja ein bisschen, wie arbeiten wir damit und wie können wir da auch reingehen.“ (T)  „Ja, wir werden das jetzt natürlich, wir werden dann jetzt über den Betreuer das regeln und so weiter, das ist klar, dass ist unsere Aufgabe. Aber das sind so die Stolpersteine. Also eigentlich für uns Kliniker ganz logische Dinge, die man einfach ausspricht und erwartet, dass dann, vielleicht dann nach kurzen Diskussionen, das gemacht wird. Funktioniert aber nicht, weil es gibt immer wieder irgendwie etwas, was dann bremst. Und auch in dieser einfachen Strukturierung: Logopäde wurde informiert, dass er das machen soll, ja geht aber nicht, weil Betreuer muss dem erst zustimmen. Weil in der Klinik war das klar, weil da der behandelnde Arzt letztendlich derjenige ist, der dann das Go gibt oder eben nicht gibt. Weil es medizinisch indiziert ist oder nicht. Aber in Außerklinik sieht es ja plötzlich ganz anders aus. Oder der Arzt muss dem zustimmen, das kann der Betreuer (nicht?), je nachdem.“ (T)  „Genau, und ich glaube, ansonsten haben wir schon auch Gesprächstermine mit den, also telefonische Gesprächstermine, steht jetzt auch die Woche noch eins an mit einem Angehörigen. Aber ich glaube auch, da merkt man auch, auch das ist so Beziehungsarbeit. Weil, ich glaube, einfach dieses Setting STUDIE für manche noch ganz neu ist. Dann so die Frage ist: „Was spielen wir überhaupt für eine Rolle in diesem Ganzen?“ Das hatte ich jetzt auch bei dem einen Angehörigen, mit dem ich telefoniert habe von Frau X. Dann, dass ich so das Gefühl habe, der will erstmal wissen, was wir eigentlich machen. Natürlich ist vorher die Aufklärung passiert, aber so dieses: „Wie sieht es denn jetzt im Alltag aus?“ Ist ja ganz normal.“ (T)  „Ich fand es sehr dienlich, auch jemand Angehörigen mit in der T- Untersuchung zu haben, um mal einen persönlichen Kontakt zu haben. Also wir haben lange besprochen. Wir haben eine Stunde, also ich sage jetzt mal, ganz basale Aufklärung über das Thema Dysphagie gemacht. Und ich hatte das Gefühl, dass es sehr wichtig war in dem Moment. Weil, der Patient ist schon ein dreiviertel Jahr auf eine Trachealkanüle, oder ein halbes Jahr auf eine Trachealkanüle, angewiesen. Und da einfach, ich glaube, dieses, bis das ankommt ist das wichtig auch, dass sich jemand dafür überhaupt zuständig fühlt, diese Aufklärungsarbeit zu leisten. Weil, ich glaube in dieser, wir müssen uns auch bewusst sein, dass in der akuten Situation der Neurorehabilitation manche Informationen einfach zu viel sind und gar nicht verarbeitet und aufgenommen werden können. Wenn man sagt: „Naja der wird die Kanüle ja wieder los, dann befasse ich mich vielleicht damit gar nicht so richtig“. Und später, wenn es dann alles gesetzt ist und das dann so eine Dauer- Situation für den ersten Moment darstellt, dann befasste ich mich erst damit und dann sind ganz viele Fragen da. Und ich glaube im ambulanten Setting fühlen sich nicht alle dafür zuständig, ist mein Eindruck, so eine intensive BASIS- Aufklärung zu machen. Weil, glaube ich, viele davon ausgehen, das hat schon die Reha gemacht oder das hat vielleicht schon mal ein Arzt gemacht.“ (T) |

*Abkürzungen.* AFNT: Ambulante Fachnachsorgeteams; T: Therapeut (Ergotherapie/Logopädie).

**Supplementary table 1 (K)**. Overview of sample statements from all code- categories [original transcript in German]

| *Thematische Code-Kategorie* | *Rollenverständnis der AFNT (Fortsetzung)* |
| --- | --- |
| Code-Subkategorie  „Ambulantes Fachnachsorgeteam (AFNT)“-  Aussagen-Beispiele  (Fortsetzung) | „Natürlich, das ist ja auch das, was wir machen, auch in unseren täglichen Gesprächen. Das machen wir ja nicht nur in den Besuchen. Aber in den täglichen Gesprächen die Überlegung, wie weit man noch eine subglottische Kanüle mit subglottischer Absaugung, obwohl jetzt auch eine Fensterkanüle geht /. Ob die Kanüle, die hat größeren Außendurchmesser als die Kanüle /. Das ist, natürlich, das ist jetzt, empfinde ich auch, das ist unsere tägliche Arbeit oder unsere Aufgabe.“ (P)  „Weil ein bestehendes System ist natürlich immer schwierig von außen mit jemanden, den man zweimal gesehen hat, dann seine kompletten Glaubenssätze gegen die Wand zu werfen, sag ich jetzt mal. Das merken wir schon, dass wir da auch oft in ein Gespräch kommen.“ (T)  „Und das ist vielleicht noch ein anderer Punkt. Dass ich auch noch ein bisschen auf der Suche bin, wie ich da auch nicht zu viel wohlwollend gute Systeme unterstütze aber auch nicht bestimmte Strukturen schwäche. Dadurch, dass es undeutlicher wird, wer für was zuständig ist zum Beispiel, genau. Das wenn sich eh schon vier Leute intensiv mit dem befassen, dann ist die Gefahr immer größer, dass, wenn da noch jemand reinkommt, dass es nur eher mehr Verwirrung gibt, und Verzögerungen.“ (A)  „Darf ich da kurz einhaken. Ich finde es, jetzt aus logopädischer Sicht, ein bisschen SELTSAM in dem Sinne, wenn ich einen externen Logopäden habe, der in eine AIP fährt, dann weiß ich eigentlich als Logopäde: „Okay, ich mache vorher die Fortbildung zum TK- Management et cetera PP“. Und wenn mir dann jemand sagen würde: „Entschuldigen Sie bitte, sie können doch eine Fortbildung machen“, würde ich mir als externer Logopäde denken: „Ja, dass mir schon klar. Ich kenn ja die Fortbildungen, die angeboten werden und ich weiß schon, dass es Fortbildungen gibt“. Also finde ich dahingehend ein bisschen schwierig vielleicht. Was anderes wäre, wenn ich selber vorschlagen würde: „Hey, wir kommen als Klinikteam und wir geben, also wir geben euch die Schulung“. Das kann ich dann eher, glaube ich, annehmen, als wenn man sie auf das Angebot hinweist, was ja sowieso jeder in Eigeninitiative tun würde. Weil, das ist bei uns Logopäden, ist das ein gängiges Prozedere, dass wir zwei bis drei Fortbildungen pro Jahr machen müssen, einfach um auf dem neusten Stand zu bleiben.“ (T)  „Das hatten wir ja schon vorhin angesprochen, ich weiß nicht WIE. Also wie wir das praktisch umsetzen sollen. Und ich finde es auch DREIST, dass wir in diesem WOHLBEHÜTETEN klinischen Setting jemanden, der wirklich draußen alleine am Patienten arbeitet, dem dann eine Empfehlung auszusprechen. Ich würde mir auch auf die Füße getreten fühlen, wenn jemand von außen sagt: „Ach, in der Klinik /. Das könnt ihr doch so und so machen, wieso macht ihr das denn nicht“?“ (A)  „Aber tatsächlich ist es so, wenn da ein Notfall in der AIP ist, wir sind nicht erreichbar, können wir gar nicht leisten, so eine rundum Notfallbetreuung.“ (A) |

*Abkürzungen.* AIP: Außerklinische Intensivpflegeteams; AFNT: Ambulante Fachnachsorgeteams; T: Therapeut (Ergotherapie/Logopädie); P: Pflegekraft und Studienassistenz; A: Arzt.

**Supplementary table 1 (L)**. Overview of sample statements from all code- categories [original transcript in German]

| **Themenfeld** | **Vertrautheitsaspekte der AFNT-Teammitglieder** |
| --- | --- |
| *Thematische Code-Kategorie* | *Ambulantes Fachnachsorgeteam (AFNT)* |
| Code-Subkategorie  „Differenzierte Behandlungszielbetrachtung“  Aussagen-Beispiel | „Dem kann ich mich ja gleich einmal anschließen. Es ist so, Ziel ist wirklich bei dem einen Patienten, dass wir Sprech-Ventil-Zeiten, Verstöpselungszeiten ausbauen. Beim anderen Patienten ist, dass man statt zwölf Mal am Tag nur elf Mal absaugt. Definitiv. Also es ist so, diese, da kann man sie in operative Ziele und strategische Ziele aufklamüstern, wie man das gerne möchte. Aber Zustandserhaltung ist auch ein Ziel.“ (P) |
| Code-Subkategorie  „Differenzierte Empfehlungsformulierung“  Aussagen-Beispiele | „Die Herausforderung ist herauszufinden, und ich glaube das haben Sie im Vorfeld so auch schon ganz gut beschrieben, welche Dokumente machen WIE Sinn. Und was kann man für diesen einen Fall wirklich Zuträgliches beitragen, genau.“ (T)  Weil wir eben diese Empfehlung gegeben haben: „Mach doch mal das, weil Entblocken noch schwierig“. Haben aber, um das Schlucken zu etablieren: „Bitte das Durchführen“. „Ja wie geht denn das?“ „Okay, wir bieten euch einen Termin an, wo wir vorbeikommen. Ganz einfache technische Geschichte aber wir machen es einmal zusammen“. Und es wurde uns gesagt, sie würden das jetzt übernehmen.“ (T) |
| Code-Subkategorie  „Interdisziplinäre Schulung“  Aussagen-Beispiel | „Ich glaube, wir haben uns schon bei uns darüber Gedanken gemacht, welche Schulungen sinnvoll sind. Wir haben auch zwei akute Themen. Also ich glaube, jetzt mit den Logopäden könnte ich manche Telefonate sogar als Schulungen einordnen. Weil wir einfach oft dann auch ganz spezifische Fall- oder auch allgemeine Themen besprechen. Wenn wir zum Beispiel darüber sprechen, warum mein Patient lieber mit entblockter Trachealkanüle Nahrung zu sich nehmen sollte. Da passiert einfach auch viel, auch in den werktäglichen Anrufen, muss man sagen. Auf der anderen Seite haben wir jetzt schon geplant für die Implementierung von einem, der Entblockung an der Beatmung, eine Schulung in einer WG durchzuführen. Und ich denke, dafür ist es dann schon gut, dass das irgendwo dokumentiert ist, wer daran dann beteiligt ist beziehungsweise wer Bescheid weiß. Andererseits glaube ich, die WG würde nicht nachgucken, sondern eher einfach im Team fragen: „Wer war denn bei der Schulung dabei?““ (T) |
| Code-Subkategorie  „Interdisziplinäre Team-Arbeit“  Aussagen-Beispiel | „Und alle Pflegekräfte reinschreiben macht zum Beispiel keinen Sinn und sehen da auch datenschutzrechtliche Probleme.“ (P) |
| Code-Subkategorie  „Kontextbasiertes Risikomanagement“  Aussagen-Beispiel | „Und wenn wir so zusagen einen Behandlungsplan geben für eine Situation, die gar nicht vorhersehbar ist, können wir sagen: „Verstöpselungszeiten ausbauen“. Dann kommen die vielleicht in eine Notfallsituation, weil wir den Impuls dazu gegeben haben. Und da ist auch viel Unsicherheit, verstehe ich, weil, die sind hier natürlich gut behütet, ich habe hier mein Reanimationsteam, wenn irgendetwas schief geht, kein Problem. Und da draußen, die müssen erstmal warten bis der Notarzt kommt.“ (A) |

*Abkürzungen.* AFNT: Ambulante Fachnachsorgeteams; T: Therapeut (Ergotherapie/Logopädie); P: Pflegekraft und Studienassistenz; A: Arzt.
